# Comparison of major depression diagnostic classification probability using the SCID, CIDI, and MINI diagnostic interviews among women in pregnancy or postpartum: An individual participant data meta‐analysis

**DOI:** 10.1002/mpr.1803

**Published:** 2019-09-30

**Authors:** Brooke Levis, Dean McMillan, Ying Sun, Chen He, Danielle B. Rice, Ankur Krishnan, Yin Wu, Marleine Azar, Tatiana A. Sanchez, Matthew J. Chiovitti, Parash Mani Bhandari, Dipika Neupane, Nazanin Saadat, Kira E. Riehm, Mahrukh Imran, Jill T. Boruff, Pim Cuijpers, Simon Gilbody, John P.A. Ioannidis, Lorie A. Kloda, Scott B. Patten, Ian Shrier, Roy C. Ziegelstein, Liane Comeau, Nicholas D. Mitchell, Marcello Tonelli, Simone N. Vigod, Franca Aceti, Rubén Alvarado, Cosme Alvarado‐Esquivel, Muideen O. Bakare, Jacqueline Barnes, Cheryl Tatano Beck, Carola Bindt, Philip M. Boyce, Adomas Bunevicius, Tiago Castro e Couto, Linda H. Chaudron, Humberto Correa, Felipe Pinheiro de Figueiredo, Valsamma Eapen, Michelle Fernandes, Barbara Figueiredo, Jane R.W. Fisher, Lluïsa Garcia‐Esteve, Lisa Giardinelli, Nadine Helle, Louise M. Howard, Dina Sami Khalifa, Jane Kohlhoff, Laima Kusminskas, Zoltán Kozinszky, Lorenzo Lelli, Angeliki A. Leonardou, Beth A. Lewis, Michael Maes, Valentina Meuti, Sandra Nakić Radoš, Purificación Navarro García, Daisuke Nishi, Daniel Okitundu Luwa E‐Andjafono, Emma Robertson‐Blackmore, Tamsen J. Rochat, Heather J. Rowe, Bonnie W.M. Siu, Alkistis Skalkidou, Alan Stein, Robert C. Stewart, Kuan‐Pin Su, Inger Sundström‐Poromaa, Meri Tadinac, S. Darius Tandon, Iva Tendais, Pavaani Thiagayson, Annamária Töreki, Anna Torres‐Giménez, Thach D. Tran, Kylee Trevillion, Katherine Turner, Johann M. Vega‐Dienstmaier, Karen Wynter, Kimberly A. Yonkers, Andrea Benedetti, Brett D. Thombs

**Affiliations:** ^1^ Lady Davis Institute for Medical Research Jewish General Hospital Montréal Québec Canada; ^2^ Department of Epidemiology, Biostatistics and Occupational Health McGill University Montréal Québec Canada; ^3^ Hull York Medical School and the Department of Health Sciences University of York York UK; ^4^ Department of Psychology McGill University Montréal Québec Canada; ^5^ Department of Psychiatry McGill University Montréal Québec Canada; ^6^ Department of Mental Health, Bloomberg School of Public Health Johns Hopkins University Baltimore MD USA; ^7^ Schulich Library of Physical Sciences, Life Sciences, and Engineering McGill University Montréal Québec Canada; ^8^ Department of Clinical, Neuro and Developmental Psychology EMGO Institute, Vrije Universiteit Amsterdam Amsterdam the Netherlands; ^9^ Department of Medicine, Department of Health Research and Policy, Department of Biomedical Data Science, Department of Statistics Stanford University Stanford CA USA; ^10^ Library Concordia University Montréal Québec Canada; ^11^ Department of Community Health Sciences, Department of Psychiatry University of Calgary Calgary Alberta Canada; ^12^ Mathison Centre for Mental Health Research & Education University of Calgary Calgary Alberta Canada; ^13^ Cuthbertson & Fischer Chair in Pediatric Mental Health University of Calgary Calgary Alberta Canada; ^14^ Department of Family Medicine McGill University Montréal Québec Canada; ^15^ Department of Medicine Johns Hopkins University School of Medicine Baltimore MD USA; ^16^ International Union for Health Promotion and Health Education, École de santé publique de l'Université de Montréal Montréal Québec Canada; ^17^ Department of Psychiatry University of Alberta Edmonton Alberta Canada; ^18^ Alberta Health Services Edmonton Alberta Canada; ^19^ Department of Medicine University of Calgary Calgary Alberta Canada; ^20^ Women's College Hospital and Research Institute University of Toronto Toronto Ontario Canada; ^21^ Department of Neurology and Psychiatry Sapienza University of Rome Rome Italy; ^22^ Escuela de Salud Pública Dr. Salvador Allende, Faculty of Medicine Universidad de Chile Santiago Chile; ^23^ Laboratorio de Investigación Biomédica, Facultad de Medicina y Nutrición Avenida Universidad Durango Mexico; ^24^ Child and Adolescent Unit Federal Neuropsychiatric Hospital Enugu Nigeria; ^25^ Childhood Neuropsychiatric Disorders Initiatives Enugu Nigeria; ^26^ Department of Psychological Sciences Birkbeck, University of London Bloomsbury London UK; ^27^ School of Nursing University of Connecticut Mansfield CT USA; ^28^ Department of Child and Adolescent Psychiatry University Medical Center Hamburg‐Eppendorf Hamburg Germany; ^29^ Discipline of Psychiatry, Westmead Clinical School, Sydney Medical School University of Sydney Sydney New South Wales Australia; ^30^ Department of Psychiatry Westmead Hospital Sydney New South Wales Australia; ^31^ Neuroscience Institute Lithuanian University of Health Sciences Kaunas Lithuania; ^32^ School of Medicine, Universidade Federal De Minas Gerais (UFMG) Belo Horizonte MG Brazil; ^33^ Departments of Psychiatry, Pediatrics, Obstetrics and Gynecology School of Medicine and Dentistry, University of Rochester Rochester NY USA; ^34^ Faculty of Medicine Universidade Federal de Minas Gerais Belo Horizonte Brazil; ^35^ Department of Neurosciences and Behavior Ribeirão Preto Medical School Ribeirão Preto Brazil; ^36^ School of Psychiatry, University of New South Wales Kensington Australia; ^37^ Ingham Institute Liverpool New South Wales Australia; ^38^ Sydney South West Local Health District Liverpool New South Wales Australia; ^39^ Faculty of Medicine, Department of Paediatrics University of Southampton and Southampton Children's Hospital Southampton UK; ^40^ The Nuffield Department of Women's & Reproductive Health, John Radcliffe Hospital and Oxford Maternal & Perinatal Health Institute, Green Templeton College University of Oxford Oxford UK; ^41^ School of Psychology University of Minho Braga Portugal; ^42^ School of Public Health and Preventive Medicine Monash University Melbourne Victoria Australia; ^43^ Perinatal Mental Health Unit CLINIC‐BCN Institut Clínic de Neurociències, Hospital Clínic Barcelona Spain; ^44^ Vulnerability, Psychopathology and Gender Research Group Generalitat de Catalunya Catalonia Spain; ^45^ August Pi i Sunyer Biomedical Research Institute Barcelona Spain; ^46^ Psychiatry Unit, Department of Health Sciences University of Florence Florence Italy; ^47^ Institute of Psychiatry, Psychology & Neuroscience King's College London London UK; ^48^ South London and Maudsley NHS Foundation Trust London UK; ^49^ Faculty of Health Sciences Ahfad University for Women Omdurman Sudan; ^50^ Department of Community Medicine, Institute of Health and Society, Faculty of Medicine University of Oslo Oslo Norway; ^51^ The Center for Global Child Health Hospital for Sick Children Toronto Ontario Canada; ^52^ Karitane Carramar New South Wales Australia; ^53^ Private Practice Hamburg Germany; ^54^ Department of Obstetrics and Gynaecology Blekinge Hospital Karlskrona Sweden; ^55^ First Department of Psychiatry, Women's Mental Health Clinic Athens University Medical School Athens Greece; ^56^ School of Kinesiology University of Minnesota Minneapolis MN USA; ^57^ Department of Psychiatry, Faculty of Medicine Chulalongkorn University Bangkok Thailand; ^58^ Impact Strategic Research Center Deakin University Geelong Victoria Australia; ^59^ Department of Psychology Catholic University of Croatia Zagreb Croatia; ^60^ Psychology Service, Regidoria de Polítiques de Gènere, Ajuntament de Terrassa Terrassa Spain; ^61^ Department of Mental Health, Graduate School of Medicine The University of Tokyo Tokyo Japan; ^62^ Department of Mental Health Policy National Institute of Mental Health, National Center of Neurology and Psychiatry Kodaira Japan; ^63^ Unité de Neuropsychologie, Département de Neurologie, Centre Neuro‐psycho‐pathologique, Faculté de Médecine Université de Kinshasa Kinshasa Democratic Republic of the Congo; ^64^ Halifax Health, Graduate Medical Education Daytona Beach FL USA; ^65^ MRC/Developmental Pathways to Health Research Unit, School of Clinical Medicine University of Witwatersrand Johannesburg South Africa; ^66^ Human and Social Development Programme Human Sciences Research Council Johannesburg South Africa; ^67^ Department of Psychiatry Castle Peak Hospital Hong Kong SAR China; ^68^ Department of Women's and Children's Health Uppsala University Uppsala Sweden; ^69^ Department of Child and Adolescent Psychiatry University of Oxford Oxford UK; ^70^ MRC/Wits Rural Public Health and Health Transitions Research Unit (Agincourt), School of Public Health, Faculty of Health Sciences University of the Witwatersrand Johannesburg South Africa; ^71^ Department of Mental Health, College of Medicine University of Malawi Zomba Malawi; ^72^ Division of Psychiatry University of Edinburgh Edinburgh UK; ^73^ College of Medicine China Medical University Taichung Taiwan; ^74^ Mind‐Body Interface Laboratory and Department of Psychiatry China Medical University Hospital Taichung Taiwan; ^75^ Department of Psychology, Faculty of Humanities and Social Sciences University of Zagreb Zagreb Croatia; ^76^ Feinberg School of Medicine Northwestern University Chicago IL USA; ^77^ Institute of Mental Health Hougang Singapore; ^78^ KK Women's and Children's Hospital Kallang Singapore; ^79^ National Healthcare Group Singapore; ^80^ Department of Emergency University of Szeged Szeged Hungary; ^81^ Epilepsy Center‐Child Neuropsychiatry Unit, ASST Santi Paolo Carlo San Paolo Hospital Milan Italy; ^82^ Facultad de Medicina Alberto Hurtado Universidad Peruana Cayetano Heredia Lima Peru; ^83^ School of Nursing and Midwifery Deakin University Melbourne Victoria Australia; ^84^ Department of Psychiatry Yale School of Medicine New Haven CT USA; ^85^ Department of Obstetrics, Gynecology, and Reproductive Sciences Yale School of Medicine New Haven CT USA; ^86^ School of Epidemiology and Public Health Yale University New Haven Connecticut USA; ^87^ Respiratory Epidemiology and Clinical Research Unit McGill University Health Centre Montréal Québec Canada; ^88^ Department of Medicine McGill University Montréal Québec Canada; ^89^ Department of Educational and Counselling Psychology McGill University Montréal Québec Canada

**Keywords:** depressive disorders, diagnostic interviews, Edinburgh Postnatal Depression Scale, individual participant data meta‐analysis, major depression

## Abstract

**Objectives:**

A previous individual participant data meta‐analysis (IPDMA) identified differences in major depression classification rates between different diagnostic interviews, controlling for depressive symptoms on the basis of the Patient Health Questionnaire‐9. We aimed to determine whether similar results would be seen in a different population, using studies that administered the Edinburgh Postnatal Depression Scale (EPDS) in pregnancy or postpartum.

**Methods:**

Data accrued for an EPDS diagnostic accuracy IPDMA were analysed. Binomial generalised linear mixed models were fit to compare depression classification odds for the Mini International Neuropsychiatric Interview (MINI), Composite International Diagnostic Interview (CIDI), and Structured Clinical Interview for DSM (SCID), controlling for EPDS scores and participant characteristics.

**Results:**

Among fully structured interviews, the MINI (15 studies, 2,532 participants, 342 major depression cases) classified depression more often than the CIDI (3 studies, 2,948 participants, 194 major depression cases; adjusted odds ratio [aOR] = 3.72, 95% confidence interval [CI] [1.21, 11.43]). Compared with the semistructured SCID (28 studies, 7,403 participants, 1,027 major depression cases), odds with the CIDI (interaction aOR = 0.88, 95% CI [0.85, 0.92]) and MINI (interaction aOR = 0.95, 95% CI [0.92, 0.99]) increased less as EPDS scores increased.

**Conclusion:**

Different interviews may not classify major depression equivalently.

## 1 INTRODUCTION

Among diagnostic interviews for classifying major depression in research, semistructured interviews, such as the Structured Clinical Interview for DSM (SCID; First, 1995), are designed to be administered by clinically trained professionals, who may insert unscripted queries and use judgement to decide whether symptoms are present. Fully structured interviews, such as the Composite International Diagnostic Interview (CIDI; Robins et al., 1988), are completely scripted and can be administered by lay interviewers. The Mini International Neuropsychiatric Interview (MINI; Lecrubier et al., 1997; Sheehan et al., 1997) is a very brief fully structured interview that was designed for rapid administration and intended to be overinclusive.

The different diagnostic interviews are typically considered equivalent for major depression classification in research (Hewitt, Gilbody, Brealey, et al., 2009; Manea, Gilbody, & McMillan, 2012; Mitchell, Meader, & Symonds, 2010; Moriarty, Gilbody, McMillan, & Manea, 2015). However, a recent individual participant data meta‐analysis (IPDMA) of 57 studies (17,158 participants) from diverse settings that controlled for participant characteristics and depressive symptom severity on the basis of the Patient Health Questionnaire‐9 (PHQ‐9) found that, among fully structured interviews, the MINI classified depression about twice as often as the CIDI. Compared with semistructured interviews, fully structured interviews (MINI excluded) classified fewer participants with high‐level depressive symptoms as depressed (Levis et al., 2018). This was the first large study to compare major depression classification across diagnostic interviews. However, it is important to determine if findings can be replicated in more than a single study.

The present study aimed to determine whether similar patterns between diagnostic interview and major depression classification could be seen among an independent set of studies that administered the Edinburgh Postnatal Depression Scale (EPDS) to women who were pregnant or had recently given birth, also using an IPDMA approach (Cox, Holden, & Sagovsky, 1987). As in the previous study, we first compared major depression classification odds within fully structured interviews to determine if different fully structured interviews perform differently (MINI vs. CIDI). Then, we compared the CIDI and MINI with the semistructured SCID, separately. In each case, we controlled for participant characteristics and depressive symptom severity on the basis of EPDS scores. Finally, we tested whether differences in classification rates between interviews were associated with depressive symptom severity.

## 2 METHODS

We used data accrued for an IPDMA on the diagnostic accuracy of the EPDS, which is the most commonly used depression screening tool for women in pregnancy or postpartum (Hewitt et al., 2009). The IPDMA was registered in PROSPERO (CRD42015024785), a protocol was published (Thombs et al., 2015), and results were reported following PRISMA‐DTA (McInnes et al., 2018) and PRISMA‐IPD (Stewart et al., 2015) reporting guidelines.

### 2.1 Identification of eligible studies

For the main IPDMA, data sets from articles in any language were eligible for inclusion if (a) they included diagnostic classification for current major depressive disorder (MDD) or major depressive episode (MDE) using any version of Diagnostic and Statistical Manual of Mental Disorders (DSM; American Psychiatric Association [APA], 1987; APA, 1994; APA, 2000) or International Classification of Diseases (ICD; World Health Organization, 1992) criteria on the basis of a validated semistructured or fully structured interview; (b) they included EPDS scores; (c) the diagnostic interview and EPDS were administered within 2 weeks of each other because DSM and ICD criteria specify that symptoms must have been present in the last 2 weeks; (d) participants were women aged ≥18 years who were not recruited from youth or college settings; and (e) participants were not recruited from psychiatric settings or because they were identified as having symptoms of depression because screening is done to identify previously unrecognised cases. For the present study, we only included studies that assessed major depression using the SCID, CIDI, and MINI because there were only three studies that used other interviews.

Data sets where not all participants were eligible were included if primary data allowed selection of eligible participants. For defining major depression, we considered MDD or MDE on the basis of the DSM or ICD. If more than one was reported, we prioritised MDE over MDD, because screening would attempt to detect depressive episodes and further interview would determine if the episode is related to MDD or bipolar disorder, and DSM over ICD.

### 2.2 Search strategy and study selection

A medical librarian searched Medline, Medline In‐Process & Other Non‐Indexed Citations and PsycINFO via OvidSP, and Web of Science via ISI Web of Knowledge from inception to June 10, 2016, using a peer‐reviewed search strategy (Methods S1; PRESS, 2016). We also reviewed reference lists of relevant reviews and queried contributing authors about non‐published studies. Search results were uploaded into RefWorks (RefWorks‐COS, Bethesda, MD, USA). After deduplication, unique citations were uploaded into DistillerSR (Evidence Partners, Ottawa, Canada) for storing and tracking search results.

Two investigators independently reviewed titles and abstracts for eligibility. If either deemed a study potentially eligible, full‐text review was done by two investigators, independently, with disagreements resolved by consensus, consulting a third investigator when necessary. A translator was consulted for determining the eligibility of one Chinese article.

### 2.3 Data extraction, contribution, and synthesis

Authors of eligible data sets were invited to contribute de‐identified primary data. We emailed corresponding authors of eligible primary studies at least three times, as necessary. If we did not receive a response, we emailed co‐authors and attempted to contact corresponding authors by phone.

Diagnostic interview used as the reference standard and country were extracted from published reports by two investigators independently, with disagreements resolved by consensus. Countries were categorised as “very high,” “high,” or “low–medium” development on the basis of the United Nation's Human Development Index, a statistical composite index that includes indicators of life expectancy, education, and income (United Nations, 2019). Participant‐level data provided in data sets included age, pregnancy status (pregnant vs. postpartum), EPDS scores, and major depression status.

Individual participant data were converted to a standard format and synthesised into a single data set with study‐level data. We compared published participant characteristics and diagnostic accuracy results with results from raw data sets and resolved any discrepancies in consultation with the original investigators. For the present study, we restricted our data to participants with complete data for all variables included in our analyses. Then, for studies that collected data at multiple time points, we restricted our data to the time point with the most participants. If there was a tie, we selected the time point with the largest number of major depression cases.

### 2.4 Statistical analyses

To isolate the association between diagnostic assessment method and major depression classification, we estimated binomial generalised linear mixed models with a logit link function. All analyses controlled for depressive symptom severity (continuous EPDS scores), age (continuous), country Human Development Index (very high, high, or low‐medium), and pregnant versus postpartum status. Given that each study only administered one diagnostic interview, these covariates were included in analyses to account for their potential influence on major depression classification. Covariates were chosen a priori on the basis of their potential influence on major depression classification as well as their availability across primary studies. To account for correlation between subjects within the same primary study, a random intercept was fit for each primary study. Fixed slopes were estimated for EPDS score, diagnostic interview, age, Human Development Index, and pregnant versus postpartum status.

We estimated generalised linear mixed models to compare major depression classification odds for MINI versus CIDI, CIDI versus SCID, and MINI versus SCID. We then fit additional models including an interaction between interview and EPDS score. All analyses were run in R using the glmer function within the lme4 package.

## 3 RESULTS

**Figure 1:**
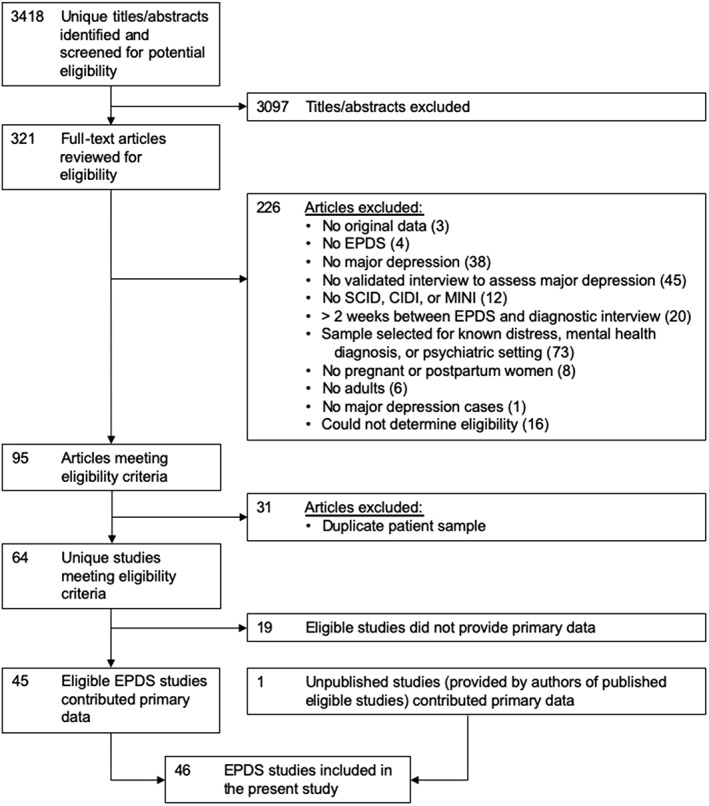
Flow diagram of study selection process

Of 3,418 unique titles and abstracts identified from the database search, 3,097 were excluded after title and abstract review and 226 were excluded after full text review, leaving 95 eligible articles with data from 64 unique participant samples, of which 45 (70% of data sets; 70% of participants) contributed data (Figure 1). Reasons for exclusion for the articles excluded at the full‐text level are given in Table S1. In addition, authors of included studies contributed data from an additional eligible study that was not identified in the search, for a total of 46 data sets. Characteristics of included studies and eligible studies that did not provide data sets are shown in Table S2. In total, 12,759 participants (1,553 [12%] with major depression) were included; none of whom were included in the previous PHQ‐9 analysis (Levis et al., 2018).

Of the 46 total included studies, there were 28 SCID studies (7,279 participants, 14% major depression), 3 CIDI studies (2,948 participants, 7% major depression), and 15 MINI studies (2,532 participants, 14% major depression; Table 1). Seventeen of the 28 SCID studies described the SCID as having been administered by clinically trained professionals.

**Table 1:** Participant data by diagnostic interview

Diagnostic interview	*N* studies	*N* participants	*N* (%) major depression
SCID	28	7,279	1,017 (14)
CIDI	3	2,948	194 (7)
MINI	15	2,532	342 (14)
Total	46	12,759	1,553 (12)

Abbreviations: CIDI, Composite International Diagnostic Interview; MINI, Mini International Neuropsychiatric Interview; SCID, Structured Clinical Interview for DSM Disorders.

**Figure 2:**
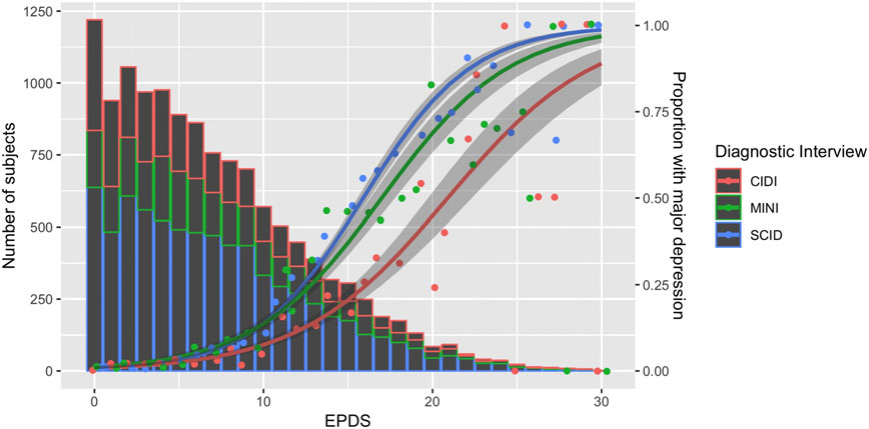
Probability of major depression classification by EPDS score for the SCID, CIDI, and MINI. CIDI, Composite International Diagnostic Interview; EPDS, Edinburgh Postnatal Depression Scale; MINI, Mini International Neuropsychiatric Interview; SCID, Structured Clinical Interview for DSM Disorders. The histogram presents the number of subjects at each EPDS score for each diagnostic interview. The lines present the proportion with major depression at each EPDS score for each diagnostic interview. The lines for each diagnostic interview were generated by estimating generalised additive logistic regression models with EPDS score as the main predictor and proportion with major depression as the outcome. The shapes of the associations were estimated directly from the data, using the mgcv package, with the amount of smoothing estimated via generalised cross validation. The analyses did not account for clustering by study

As shown in Figure 2 and Table S3, for all interviews, the proportion with major depression generally increased as EPDS scores increased.

Model coefficients for each analysis are shown in Table S4. Among fully structured interviews, controlling for EPDS scores, the MINI was more likely to classify major depression than the CIDI (adjusted odds ratio [aOR] = 3.72; 95% confidence interval [CI] [1.21, 11.43]). The CIDI and MINI tended to classify major depression less often than the SCID, but there was high uncertainty in estimates (aOR for CIDI vs. SCID = 0.34, 95% CI [0.09, 1.34]; aOR for MINI vs. SCID = 0.91, 95% CI [0.43, 1.94]).

As EPDS scores increased, the probability of diagnosis increased more for the MINI than for the CIDI (interaction aOR = 1.07, 95% CI [1.03, 1.12]) but increased less for both the CIDI and MINI than for the SCID (interaction aOR for CIDI = 0.88, 95% CI [0.85, 0.92]; interaction aOR for MINI = 0.95, 95% CI [0.92, 0.99]).

## 4 DISCUSSION

We compared depression classification across diagnostic interviews in studies that administered the EPDS with women in pregnancy or postpartum, controlling for participant characteristics and depressive symptom severity on the basis of EPDS scores. Among fully structured interviews, odds of major depression were substantially higher for the MINI than the CIDI. As depressive symptom severity increased, the probability of diagnosis increased more for the MINI than for the CIDI. There were no definitive differences in classification odds between the CIDI and SCID and between the MINI and SCID, but, as EPDS scores increased, likelihood of classification increased less for the CIDI and MINI than for the SCID. Results were similar to those of our previous study that assessed depressive symptom severity in diverse patient groups with the PHQ‐9 (Levis et al., 2018). In that study, on the basis of subgroup analyses by PHQ‐9 scores, we found that the CIDI classified fewer participants with high‐level depressive symptoms as depressed than the SCID. Due to limited numbers of participants and major depression cases for each interview across EPDS scores in the present study, we were unable to conduct subgroup analyses based on EPDS scores. However, our interaction analyses were generally consistent with previous findings.

There are limitations to consider. First, we were unable to obtain primary data for 19 of 64 eligible data sets identified in our search (30% of data sets; 30% of participants). Second, only three included studies used the CIDI, one of which had only one major depression case. Third, across interviews, there were few participants with high EPDS scores and few major depression cases with low EPDS scores. For the CIDI, data were sparse across EPDS scores. Notwithstanding, the previous PHQ‐9 study and the present study have used samples many times the size of other studies that have attempted to compare diagnostic interviews for major depression (Anthony et al., 1985; Booth, Kirchner, Hamilton, Harrell, & Smith, 1998; Brugha, Jenkins, Taub, Meltzer, & Bebbington, 2001; Hesselbrock, Stabenau, Hesselbrock, Mirkin, & Meyer, 1982; Jordanova, Wickramesinghe, Gerada, & Prince, 2004). Fourth, residual confounding may exist. We were only able to consider variables collected in the original investigations, and the included study‐level variables may not apply uniformly to all participants in a study. Finally, not all SCID studies described interviewer qualifications. It is possible that use of untrained interviewers may have reduced performance differences across interviews.

## 5 CONCLUSION

The previous PHQ‐9 IPDMA found that different diagnostic interviews may not be equivalent for major depression classification. In the present study, we observed similar patterns. The CIDI and MINI were designed as less resource‐intensive options that can be administered by research staff without diagnostic skills, but they may misclassify major depression in substantial numbers of patients compared with the SCID. The findings of both the previous and present IPDMAs suggest that different interviews may not classify major depression equivalently and should be combined in meta‐analyses with caution.

## DATA ACCESSIBILITY

Requests to access data should be made to the corresponding author at brett.thombs@mcgill.ca.

## DECLARATION OF INTEREST STATEMENT

All authors have completed the ICMJE uniform disclosure form and declare no support from any organisation for the submitted work; no financial relationships with any organisations that might have an interest in the submitted work in the previous 3 years with the following exceptions: Dr. Patten reports grants from Hotchkiss Brain Institute/Pfizer Competition, outside the submitted work. Dr. Tonelli declares that he has received a grant from Merck Canada, outside the submitted work. Dr. Vigod declares that she receives royalties from UpToDate, outside the submitted work. Dr. Beck declares that she receives royalties for her Postpartum Depression Screening Scale published by Western Psychological Services. Dr. Boyce declares that he receives grants and personal fees from Servier, grants from Lundbeck, and personal fees from AstraZeneca, all outside the submitted work. Dr. Howard declares that she has received personal fees from NICE Scientific Advice, outside the submitted work. Dr. Nishi declares that he has received personal fees from Sumitomo Dainippon Pharma Co., Ltd., outside the submitted work. Dr. Sundström‐Poromaa declares that she has served on advisory boards and acted as invited speaker at scientific meetings for MSD, Novo Nordisk, Bayer Health Care, and Lundbeck A/S. Dr. Yonkers declares that she receives royalties from UpToDate, outside the submitted work.

## AUTHOR CONTRIBUTIONS

B. L., D. M., J. T. B., P. C., S. G., J. P. A. I., L. A. K., S. B. P., I. S., R. C. Z., L. C., N. D. M., M. T., S. N. V., A. Benedetti, and B. D. T. were responsible for the study conception and design. J. T. B. and L. A. K. designed and conducted database searches to identify eligible studies. F. A., R. A., C. A. E., M. O. B., J. B., C. T. B., C. B., P. M. Boyce, A. Bunevicius, T. C. e. C., L. H.C., H. C., F. P. F., V. E., M. F., B. F., J. R. W. F., L. G. E., L. G., N. H., L. M. H., D. S. K., J. K., L. K., Z. K., L. L., A. A. L., B. A. L., M. M., V. M., S. N. R., P. N. G., D. Nishi, D. O. L. E. A., E. R. B., T. J. R., H. J. R., B. W. M. S., A. Skalkidou, A. Stein, R. C. S., K. P. S., I. S. P., M. T., S. D. T., I. T., P. T., A. T., A. T. G., T. D. T., K. Trevillion, K. Turner, J. M. V. D., K. W., and K. A. Y. contributed primary data sets that were included in this study. B. L., Y. S., C. H., D. B. R., A. K., Y. W., M. A., T. A. S., M. J. C., P. M. Bhandari, D. Neupane, N. S., K. E. R., and M. I. contributed to data extraction and coding for the meta‐analysis. B. L., A. Benedetti, and B. D. T. contributed to the data analysis and interpretation. B. L., D. M., A. Benedetti, and B. D. T. contributed to drafting the manuscript. All authors provided a critical review and approved the final manuscript. A. Benedetti and B. D. T. are the guarantors; they had full access to all the data in the study and take responsibility for the integrity of the data and the accuracy of the data analyses.

## Supporting information

Data S1. Supporting Information
Methods S1. Complete Search Strategies
Table S1. Reasons for Exclusion at Full‐text Level (*N* = 226)
Table S2a. Characteristics of Included Primary Studies (*N* = 46)
Table S2b. Characteristics of Eligible Primary Studies That Did Not Provide Data for the Present Study (*N* = 19)
Table S3. Number and Proportion of Participants with Major Depression at each EPDS Score for the Structured Clinical Interview for DSM Disorders, Composite International Diagnostic Interview, and Mini Neurospsychiatric Diagnostic Interview
Table S4a. Estimates of Fixed Effects from Model Comparing the Mini Neurospsychiatric Diagnostic Interview to the Composite International Diagnostic Interview
Table S4b. Estimates of Fixed Effects from Model Comparing the Composite International Diagnostic Interview to the Structured Clinical Interview for DSM Disorders
Table S4c**.** Estimates of Fixed Effects from Model Comparing the Mini Neurospsychiatric Diagnostic Interview to the Structured Clinical Interview for DSM Disorders
Table S4d. Estimates of Fixed Effects from Model Comparing the Mini Neurospsychiatric Diagnostic Interview (MINI) to the Composite International Diagnostic Interview, Including an Interaction between MINI and Depressive Symptom Severity based on the Edinburgh Postnatal Depression Scale
Table S4e. Estimates of Fixed Effects from Model Comparing the Composite International Diagnostic Interview (CIDI) to the Structured Clinical Interview for DSM Disorders, Including an Interaction between CIDI and Depressive Symptom Severity based on the Edinburgh Postnatal Depression Scale
Table S4f. Estimates of Fixed Effects from Model Comparing the the Mini Neurospsychiatric Diagnostic Interview (MINI) to the Structured Clinical Interview for DSM Disorders, Including an Interaction between MINI and Depressive Symptom Severity based on the Edinburgh Postnatal Depression Scale
