# Supplementary material for: Comparison of major depression diagnostic classification probability using the SCID, CIDI, and MINI diagnostic interviews among women in pregnancy or postpartum: An individual participant data meta‐analysis
Source: Int J Methods Psychiatr Res. 2019 Sep 30;28(4):e1803. doi: 10.1002/mpr.1803 (PMC7027670; doi:10.1002/mpr.1803)
Supplement: Supplementary file 1 — Data S1. Supporting Information Methods S1. Complete Search Strategies Table S1. Reasons for Exclusion at Full‐text Level (N = 226) Table S2a. Characteristics of Included Primary Studies (N = 46) Table S2b. Characteristics of Eligible Primary Studies That Did Not Provide Data for the Present Study (N = 19) Table S3. Number and Proportion of Participants with Major Depression at each EPDS Score for the Structured Clinical Interview for DSM Disorders, Composite International Diagnostic Interview, and Mini Neurospsychiatric Diagnostic Interview Table S4a. Estimates of Fixed Effects from Model Comparing the Mini Neurospsychiatric Diagnostic Interview to the Composite International Diagnostic Interview Table S4b. Estimates of Fixed Effects from Model Comparing the Composite International Diagnostic Interview to the Structured Clinical Interview for DSM Disorders Table S4c. Estimates of Fixed Effects from Model Comparing the Mini Neurospsychiatric Diagnostic Interview to the Structured Clinical Interview for DSM Disorders Table S4d. Estimates of Fixed Effects from Model Comparing the Mini Neurospsychiatric Diagnostic Interview (MINI) to the Composite International Diagnostic Interview, Including an Interaction between MINI and Depressive Symptom Severity based on the Edinburgh Postnatal Depression Scale Table S4e. Estimates of Fixed Effects from Model Comparing the Composite International Diagnostic Interview (CIDI) to the Structured Clinical Interview for DSM Disorders, Including an Interaction between CIDI and Depressive Symptom Severity based on the Edinburgh Postnatal Depression Scale Table S4f. Estimates of Fixed Effects from Model Comparing the the Mini Neurospsychiatric Diagnostic Interview (MINI) to the Structured Clinical Interview for DSM Disorders, Including an Interaction between MINI and Depressive Symptom Severity based on the Edinburgh Postnatal Depression Scale [file MPR-28-e1803-s001.docx]

**Supplementary Material**

**Supplementary Methods 1.** Complete Search Strategies

**Supplementary Table 1.** Reasons for Exclusion at Full-text Level (N = 226)

**Supplementary Table 2a.** Characteristics of Included Primary Studies (N = 46)

**Supplementary Table 2b.** Characteristics of Eligible Primary Studies That Did Not Provide Data for the Present Study (N = 19)

**Supplementary Table 3.** Number and Proportion of Participants with Major Depression at each EPDS Score for the Structured Clinical Interview for DSM Disorders, Composite International Diagnostic Interview, and Mini Neurospsychiatric Diagnostic Interview

**Supplementary Table 4a.** Estimates of Fixed Effects from Model Comparing the Mini Neurospsychiatric Diagnostic Interview to the Composite International Diagnostic Interview

**Supplementary Table 4b.** Estimates of Fixed Effects from Model Comparing the Composite International Diagnostic Interview to the Structured Clinical Interview for DSM Disorders

**Supplementary Table 4c.** Estimates of Fixed Effects from Model Comparing the Mini Neurospsychiatric Diagnostic Interview to the Structured Clinical Interview for DSM Disorders

**Supplementary Table 4d.** Estimates of Fixed Effects from Model Comparing the Mini Neurospsychiatric Diagnostic Interview (MINI) to the Composite International Diagnostic Interview, Including an Interaction between MINI and Depressive Symptom Severity based on the Edinburgh Postnatal Depression Scale

**Supplementary Table 4e.** Estimates of Fixed Effects from Model Comparing the Composite International Diagnostic Interview (CIDI) to the Structured Clinical Interview for DSM Disorders, Including an Interaction between CIDI and Depressive Symptom Severity based on the Edinburgh Postnatal Depression Scale

**Supplementary Table 4f.** Estimates of Fixed Effects from Model Comparing the the Mini Neurospsychiatric Diagnostic Interview (MINI) to the Structured Clinical Interview for DSM Disorders, Including an Interaction between MINI and Depressive Symptom Severity based on the Edinburgh Postnatal Depression Scale

**Supplementary Methods 1. Complete Search Strategies**

**MEDLINE (OvidSP)**

1. EPDS.af.

2. Edinburgh Postnatal Depression.af.

3. Edinburgh Depression Scale.af.

4. or/1-3

5. Mass Screening/

6. Psychiatric Status Rating Scales/

7. "Predictive Value of Tests"/

8. "Reproducibility of Results"/

9. exp "Sensitivity and Specificity"/

10. Psychometrics/

11. Prevalence/

12. Reference Values/

13. Reference Standards/

14. exp Diagnostic Errors/

15. Mental Disorders/di, pc [Diagnosis, Prevention & Control]

16. Mood Disorders/di, pc [Diagnosis, Prevention & Control]

17. Depressive Disorder/di, pc [Diagnosis, Prevention & Control]

18. Depressive Disorder, Major/di, pc [Diagnosis, Prevention & Control]

19. Depression, Postpartum/di, pc [Diagnosis, Prevention & Control]

20. Depression/di, pc [Diagnosis, Prevention & Control]

21. validation studies.pt.

22. comparative study.pt.

23. screen*.af.

24. prevalence.af.

25. predictive value*.af.

26. detect*.ti.

27. sensitiv*.ti.

28. valid*.ti.

29. revalid*.ti.

30. predict*.ti.

31. accura*.ti.

32. psychometric*.ti.

33. identif*.ti.

34. specificit*.ab.

35. cut?off*.ab.

36. cut* score*.ab.

37. cut?point*.ab.

38. threshold score*.ab.

39. reference standard*.ab.

40. reference test*.ab.

41. index test*.ab.

42. gold standard.ab.

43. or/5-42

44. 4 and 43

**PsycINFO (OvidSP)**

1. EPDS.af.

2. Edinburgh Postnatal Depression.af.

3. Edinburgh Depression Scale.af.

4. or/1-3

5. Diagnosis/

6. Medical Diagnosis/

7. Psychodiagnosis/

8. Misdiagnosis/

9. Screening/

10. Health Screening/

11. Screening Tests/

12. Prediction/

13. Cutting Scores/

14. Psychometrics/

15. Test Validity/

16. screen*.af.

17. predictive value*.af.

18. detect*.ti.

19. sensitiv*.ti.

20. valid*.ti.

21. revalid*.ti.

22. accura*.ti.

23. psychometric*.ti.

24. specificit*.ab.

25. cut?off*.ab.

26. cut* score*.ab.

27. cut?point*.ab.

28. threshold score*.ab.

29. reference standard*.ab.

30. reference test*.ab.

31. index test*.ab.

32. gold standard.ab.

33. or/5-32

34. 4 and 33

**Web of Science (Web of Knowledge)**

#1. TS=(EPDS OR “Edinburgh Postnatal Depression” OR “Edinburgh Depression Scale”)

#2. TS=(screen* OR prevalence OR “predictive value*” OR detect* OR sensitiv* OR valid* OR revalid* OR predict* OR accura* OR psychometric* OR identif* OR specificit* OR cutoff* OR “cut off*” OR “cut* score*” OR cutpoint* OR “cut point*” OR “threshold score*” OR “reference standard*” OR “reference test*” OR “index test*” OR “gold standard” OR “reliab*”)

#2 AND #1

*Databases=SCI-EXPANDED, SSCI, A&HCI*

**Supplementary Table 1. Reasons for Exclusion at Full-text Level (N = 226)**

| **Reference** | **Reason For Exclusion** |
| --- | --- |
| Abiodun OA. Postnatal depression in primary care populations in Nigeria. Gen Hosp Psychiatry. 2006;**28**:133-6. | Could not determine eligibility^a^ |
| Abou-Saleh MT, Ghubash R, Karim L, Krymski M, Bhai I. Hormonal aspects of postpartum depression. Psychoneuroendocrinology. 1998;**23**:465-75. | > 2 weeks between EPDS and diagnostic interview |
| Aceti F, Baglioni V, Ciolli P, De Bei F, Di Lorenzo F, Ferracuti S, et al. Maternal attachment patterns and personality in post partum depression. Riv Psichiatr. 2012;**47**:214-20. | Sample selected for known distress, mental health diagnosis, or psychiatric setting |
| Adewuya AO, Eegunranti AB, Lawal AM. Prevalence of postnatal depression in Western Nigerian women: a controlled study. Int J Psychiatry Clin Pract. 2005;**9**:60-4. | Could not determine eligibility^a^ |
| Adewuya AO. Early postpartum mood as a risk factor for postnatal depression in Nigerian women. Am J Psychiatry. 2006;**163**:1435-7. | No validated interview to assess major depression |
| Ahn S, Corwin EJ. The association between breastfeeding, the stress response, inflammation, and postpartum depression during the postpartum period: Prospective cohort study. Int J Nurs Stud. 2015;**52**:1582-90. | Major depression not assessed |
| Alami KM, Kadri N, Berrada S. Prevalence and psychosocial correlates of depressed mood during pregnancy and after childbirth in a Moroccan sample. Arch Womens Ment HealthArch Womens Ment Health. 2006;**9**:343-6. | Could not determine eligibility^a^ |
| Albacar G, Sans T, MartinSantos R, GarciaEsteve L, Guillamat R, Sanjuan J, et al. Thyroid function 48 h after delivery as a marker for subsequent postpartum depression. Psychoneuroendocrinology. 2010;**35**:738-42. | Sample selected for known distress, mental health diagnosis, or psychiatric setting |
| Albacar G, Sans T, MartinSantos R, GarciaEsteve L, Guillamat R, Sanjuan J, et al. An association between plasma ferritin concentrations measured 48h after delivery and postpartum depression. J Affect DisordJ Affect Disord. 2011;**131**:136-42. | Sample selected for known distress, mental health diagnosis, or psychiatric setting |
| Alexander S, Palmer C, Stone PC. Evaluation of screening instruments for depression and anxiety in breast cancer survivors. Breast Cancer Res Treat. 2010;**122**:573-8. | No pregnant or postpartum women |
| Algul A, Semiz UB, Dundar O, Ates MA, Basoglu C, Ebrinc S, et al. Psychosocial and hormone related risk factors for early postnatal depressive symptoms in Turkish women. Neurol Psychiat Br. 2008;**15**:117-22. | Major depression not assessed |
| Al-Modayfer O, Alatiq Y, Khair O, Abdelkawi S. Postpartum depression and related risk factors among Saudi females. Int J Cult Ment Health. 2015;**8**:316-24. | No validated interview to assess major depression |
| Alvarado-Esquivel C, Sifuentes-Alvarez A, Estrada-Martínez S, Salas-Martínez C, Hernndez-Alvarado AB, Ortiz-Rocha SG, et al. Prevalence of postnatal depression in women attending public hospitals in Durango, Mexico. Gac Med Mex. 2010;**146**:1-9. | No validated interview to assess major depression |
| Alvarado-Esquivel C, Sifuentes-Alvarez A, Salas-Martinez C. Unhappiness with the Fetal Gender is associated with Depression in Adult Pregnant Women Attending Prenatal Care in a Public Hospital in Durango, Mexico. Int J Biomed Sci. 2016;**12**:36-41. | Sample selected for known distress, mental health diagnosis, or psychiatric setting |
| Areias ME, Kumar R, Barros H, Figueiredo E. Comparative incidence of depression in women and men, during pregnancy and after childbirth. Validation of the Edinburgh Postnatal Depression Scale in Portuguese mothers. Br J Psychiatry. 1996;**169**:30-5. | No validated interview to assess major depression |
| Areias ME, Kumar R, Barros H, Figueiredo E. Correlates of postnatal depression in mothers and fathers. Br J Psychiatry. 1996;**169**:36-41. | No validated interview to assess major depression |
| Austin MP, Dudley M, Launders C, Dixon C, Macartney-Bourne F. Description and evaluation of a domiciliary perinatal mental health service focussing on early intervention. Arch Womens Ment Health. 1999;**2**:169-73. | Sample selected for known distress, mental health diagnosis, or psychiatric setting |
| Austin MP, Frilingos M, Lumley J, Hadzi-Pavlovic D, Roncolato W, Acland S, et al. Brief antenatal cognitive behaviour therapy group intervention for the prevention of postnatal depression and anxiety: a randomised controlled trial. J Affect Disord. 2008;**105**:35-44. | Sample selected for known distress, mental health diagnosis, or psychiatric setting |
| Austin MP, Hadzi-Pavlovic D, Priest SR, Reilly N, Wilhelm K, Saint K, Parker G. Depressive and anxiety disorders in the postpartum period: how prevalent are they and can we improve their detection? Arch Womens Ment Health. 2010;**13**:395-401. | Major depression not assessed |
| Austin MP, Hadzi-Pavlovic D, Saint K, Parker G. Antenatal screening for the prediction of postnatal depression: validation of a psychosocial Pregnancy Risk Questionnaire. Acta Psychiatr Scand. 2005;**112**:310-7. | Major depression not assessed |
| Azar R, Paquette D, Zoccolillo M, Baltzer F, Tremblay RE. The association of major depression, conduct disorder, and maternal overcontrol with a failure to show a cortisol buffered response in 4-month-old infants of teenage mothers. Biological Psychiatry. 2007;**62**:573-9. | Not a sample of adults |
| [Bågedahl‐Strindlund](https://onlinelibrary.wiley.com/action/doSearch?ContribAuthorStored=B%C3%A5gedahl-Strindlund%2C+M) M, [Monsen Börjesson](https://onlinelibrary.wiley.com/action/doSearch?ContribAuthorStored=B%C3%B6rjesson%2C+K+Monsen) K. Postnatal depression: a hidden illness. Acta Psychiatr Scand. 1998;**98**:272-5. | Sample selected for known distress, mental health diagnosis, or psychiatric setting |
| Barnett B, Matthey S, Gyaneshwar R. Screening for postnatal depression in women of non-English speaking background. Arch Womens Ment Health. 1999;**2**:67-74. | Did not administer the SCID, CIDI, or MINI |
| Bergant AM, Heim K, Ulmer H, Illmensee K. Early postnatal depressive mood: associations with obstetric and psychosocial factors. J Psychosom Res. 1999;**46**:391-4. | Major depression not assessed |
| Bergant AM, Nguyen T, Heim K, Ulmer H, Dapunt O. German language version and validation of the Edinburgh postnatal depression scale. Dtsch Med Wochenschr. 1998;**123**:35-40. | No validated interview to assess major depression |
| Bick DE, MacArthur C, Lancashire RJ. What influences the uptake and early cessation of breast feeding? Midwifery. 1998;**14**:242-7. | Major depression not assessed |
| Bloch M, Rotenberg N, Koren D, Klein E. Risk factors associated with the development of postpartum mood disorders. J Affect Disord. 2005;**88**:9-18. | > 2 weeks between EPDS and diagnostic interview |
| Boath E, Cox J, Lewis M, Jones P, Pryce A. When the cradle falls: the treatment of postnatal depression in a psychiatric day hospital compared with routine primary care. J Affect Disord. 1999;**53**:143-51. | Sample selected for known distress, mental health diagnosis, or psychiatric setting |
| Boyce P, Hickey A. Psychosocial risk factors to major depression after childbirth. Soc Psychiatry Psychiatr Epidemiol. 2005;**40**:605-12. | Sample selected for known distress, mental health diagnosis, or psychiatric setting |
| Boyce P, Stubbs J, Todd A. The Edinburgh Postnatal Depression Scale: validation for an Australian sample. Aust N Z J Psychiatry. 1993;**27**:472-6. | Sample selected for known distress, mental health diagnosis, or psychiatric setting |
| Browne JC, Scott KM, Silvers KM. Fish consumption in pregnancy and omega-3 status after birth are not associated with postnatal depression. J Affect Disord. 2006;**90**:131-9. | Sample selected for known distress, mental health diagnosis, or psychiatric setting |
| Brugha TS, Wheatley S, Taub NA, Culverwell A, Friedman T, Kirwan P, et al. Pragmatic randomized trial of antenatal intervention to prevent post-natal depression by reducing psychosocial risk factors. Psychol Med. 2000;**30**:1273-81. | Sample selected for known distress, mental health diagnosis, or psychiatric setting |
| Bunevičius A, Kusminskas L, Bunevičius R. Validation of the Lithuanian version of the Edinburgh Postnatal Depression Scale. Med Lith. 2009;**45**:544. | No validated interview to assess major depression |
| Bunevicius A, Kusminskas L, Bunevicius R. Validity of the Edinburgh Postnatal Depression Scale. Eur Psychiatry. 2009;**24:**S896. | No validated interview to assess major depression |
| Burns A, O'Mahen H, Baxter H, Bennert K, Wiles N, Ramchandani P, et al. A pilot randomised controlled trial of cognitive behavioural therapy for antenatal depression. BMC Psychiatry. 2013;**13**:33. | Sample selected for known distress, mental health diagnosis, or psychiatric setting |
| Byatt N, Biebel K, Simas TAM, Sarvet B, Ravech M, Allison J, Straus J. Improving perinatal depression care: The Massachusetts Child Psychiatry Access Project for Moms. Gen Hosp Psychiatry. 2016;**40**:12-7. | Major depression not assessed |
| Caramlau I, Barlow J, Sembi S, McKenzie-McHarg K, McCabe C. Mums 4 Mums: structured telephone peer-support for women experiencing postnatal depression. Pilot and exploratory RCT of its clinical and cost effectiveness. Trials . 2011;**12**:88. | No original data |
| Carothers AD, Murray L. Estimating psychiatric morbidity by logistic regression: application to post-natal depression in a community sample. Psychol Med. 1990;**20**:695-702. | No validated interview to assess major depression |
| Carpiniello B, Pariante CM, Serri F, Costa G, Carta MG. Validation of the Edinburgh Postnatal Depression Scale in Italy. J Psychosom Obstet Gynecol. 1997;**18**:280-5. | No validated interview to assess major depression |
| [Castañón](https://europepmc.org/search?query=AUTH:%22Casta%C3%B1%C3%B3n+S+C%22&page=1) SC, Pinto LJ. Use of the Edinburgh Postnatal Depression Scale to detect postpartum depression. Rev Med Chil. 2008;**136**:851-8. | Sample selected for known distress, mental health diagnosis, or psychiatric setting |
| Chaudron LH, Nirodi N. The obsessive-compulsive spectrum in the perinatal period: a prospective pilot study. Arch Womens Ment Health. 2010;**13**:403-10. | > 2 weeks between EPDS and diagnostic interview |
| Chee CY, Chong YS, Ng TP, Lee DT, Tan LK, Fones CS. The association between maternal depression and frequent non-routine visits to the infant's doctor--a cohort study. J Affect Disord. 2008;**107**:247-53. | Sample selected for known distress, mental health diagnosis, or psychiatric setting |
| Chee CYI, Lee DTS, Chong YS, Tan LK, Ng TR, Fones CSL. Confinement and other psychosocial factors in perinatal depression: A transcultural study in Singapore. J Affect Disord. 2005;**89**:157-66. | Sample selected for known distress, mental health diagnosis, or psychiatric setting |
| Chen H, Bautista D, Ch'ng YC, Li W, Chan E, Rush AJ. Screening for postnatal depression in Chinese-speaking women using the Hong Kong translated version of the Edinburgh Postnatal Depression Scale. Asia Pac Psychiatry. 2013;**5**:E64-E72. | No validated interview to assess major depression |
| Chibanda D, Verhey R, Gibson LJ, Munetsi E, Machando D, Rusakaniko S, et al. Validation of screening tools for depression and anxiety disorders in a primary care population with high HIV prevalence in Zimbabwe. J Affect Disord. 2016;**198**:50-55. | EPDS not administered |
| Clarke PJ. Validation of two postpartum depression screening scales with a sample of First Nations and Metis women. Can J Nurs Res. 2008;**40**:112-25. | Major depression not assessed |
| Class QA, Verhulst J, Heiman JR. Exploring the heterogeneity in clinical presentation and functional impairment of postpartum depression. J Reprod Infant Psychol. 2013;**31**:183-94 | Sample selected for known distress, mental health diagnosis, or psychiatric setting |
| Clifford C, Day A, Cox J, Werrett J. A cross-cultural analysis of the use of the Edinburgh Post-Natal Depression Scale (EPDS) in health visiting practice. J Adv Nurs. 1999;**30**:655-64. | No validated interview to assess major depression |
| Coleman R, Morison L, Paine K, Powell RA, Walraven G. Women's reproductive health and depression: a community survey in the Gambia, West Africa. Soc Psychiatry Psychiatr Epidemiol. 2006;**41**:720-7. | No validated interview to assess major depression |
| Cooper PJ, Murray L, Wilson A, Romaniuk H. Controlled trial of the short- and long-term effect of psychological treatment of post-partum depression. I. Impact on maternal mood. Br J Psychiatry. 2003;**182**:412-9. | Sample selected for known distress, mental health diagnosis, or psychiatric setting |
| Costas J, Gratacòs M, Escaramís G, Martín-Santos R, de Diego Y, Baca- García E, et al. Association study of 44 candidate genes with depressive and anxiety symptoms in post-partum women. J Psychiatr Res. 2010;**44**:717-24. | Sample selected for known distress, mental health diagnosis, or psychiatric setting |
| Cox JL, Chapman G, Murray D, Jones P. Validation of the Edinburgh Postnatal Depression Scale (EPDS) in non-postnatal women. J Affect Disord. 1996;**39**:185-9. | No validated interview to assess major depression |
| Cox JL, Holden JM, Sagovsky R. Detection of postnatal depression. Development of the 10-item Edinburgh Postnatal Depression Scale. Br J Psychiatry. 1987;**150**:782-6. | Sample selected for known distress, mental health diagnosis, or psychiatric setting |
| Cox JL, Murray D, Chapman G. A controlled study of the onset, duration and prevalence of postnatal depression. Br J Psychiatry. 1993;**163**:27-31. | No validated interview to assess major depression |
| Crotty F, Sheehan J. Prevalence and detection of postnatal depression in an Irish community sample. Irish Journal of Psychol Med. 2004;**21**:117-21. | Did not administer the SCID, CIDI, or MINI |
| de Souza Ribeiro Martins C, dos Santos Motta JV, Quevedo LA, de Matos MB, Pinheiro KAT, de Mattos Souza LD, et al. Comparison of two instruments to track depression symptoms during pregnancy in a sample of pregnant teenagers in Southern Brazil. J Affect Disord. 2015;**177**:95-100. | Not a sample of adults |
| Dennis CL, Hodnett E, Kenton L, Weston J, Zupancic J, Stewart DE, Kiss A. Effect of peer support on prevention of postnatal depression among high risk women: multisite randomised controlled trial. BMJ. 2009;**338**:a3064. | Sample selected for known distress, mental health diagnosis, or psychiatric setting |
| Ebeigbe PN, Akhigbe KO. Incidence and associated risk factors of postpartum depression in a tertiary hospital in Nigeria. Niger Postgrad Med J. 2008;**15**:15-8. | Major depression not assessed |
| Eberhard-Gran M, Eskild A, Tambs K, Schei B, Opjordsmoen S. The Edinburgh Postnatal Depression Scale: validation in a Norwegian community sample. Nord J Psychiatry. 2001;**55**:113-7. | No validated interview to assess major depression |
| Ekeroma AJ, Ikenasio-Thorpe B, Weeks S, Kokaua J, Puniani K, Stone P, Foliaki SA. Validation of the Edinburgh Postnatal Depression Scale (EPDS) as a screening tool for postnatal depression in Samoan and Tongan women living in New Zealand. N Z M J. 2012;**125**:41-9. | > 2 weeks between EPDS and diagnostic interview |
| Ekuklu G, Tokuc B, Eskiocak M, Berberoglu U, Saltik A. Prevalence of postpartum depression in Edirne, Turkey, and related factors. J Reprod Med. 2004;**49**:908-14. | Major depression not assessed |
| El-Ibiary SY, Hamilton SP, Abel R, Erdman CA, Robertson PA, Finley PR. A pilot study evaluating genetic and environmental factors for postpartum depression. Innov Clin Neurosci. 2013;**10**:15-22. | Sample selected for known distress, mental health diagnosis, or psychiatric setting |
| Elliott SA, Leverton TJ, Sanjack M, Turner H, Cowmeadow P, Hopkins J, Bushnell D. Promoting mental health after childbirth: a controlled trial of primary prevention of postnatal depression. Br J Clin Psychol. 2000;**39**:223-41. | Sample selected for known distress, mental health diagnosis, or psychiatric setting |
| Fairbrother N, Young AH, Janssen P, Antony MM, Tucker E. Depression and anxiety during the perinatal period. BMC Psychiatry. 2015;**15**:206. | Sample selected for known distress, mental health diagnosis, or psychiatric setting |
| Farhat A, Saeidi R, Mohammadzadeh A, Hesari H. Prevalence of Postpartum Depression; a longitudinal study. Iran J Neonatol. 2015;**6**:39-44. | Major depression not assessed |
| Felice E, Saliba J, Grech V, Cox J. Prevalence rates and psychosocial characteristics associated with depression in pregnancy and postpartum in Maltese women. J Affect Disord. 2004;**82**:297-301. | Did not administer the SCID, CIDI, or MINI |
| Felice E, Saliba J, Grech V, Cox J. Validation of the Maltese version of the Edinburgh Postnatal Depression Scale. Arch Womens Ment Health. 2006;9(2):75-80. | Did not administer the SCID, CIDI, or MINI |
| Flynn HA, Sexton M, Ratliff S, Porter K, Zivin K. Comparative performance of the Edinburgh Postnatal Depression Scale and the Patient Health Questionnaire-9 in pregnant and postpartum women seeking Psychiatr Serv. Psychiatry Res. 2011;**187**:130-4. | Sample selected for known distress, mental health diagnosis, or psychiatric setting |
| Gallanti AME, Rodríguez CEAM, Rodríguez IM, Sosa MA. Puerperal depression and its association with demographic and social factors, the way of resolution of pregnancy and the newborn clinical evolution. Medula. 2015;**24**:25-34. | Major depression not assessed |
| Gelabert E, Subira S, Plaza A, Torres A, Navarro P, Imaz ML, et al. The Vulnerable Personality Style Questionnaire: psychometric properties in Spanish postpartum women. Arch Womens Ment Health. 2011;**14**:115-24. | Sample selected for known distress, mental health diagnosis, or psychiatric setting |
| Gemmill AW, Leigh B, Ericksen J, Milgrom J. A survey of the clinical acceptability of screening for postnatal depression in depressed and non-depressed women. BMC Public Health. 2006;**6**:211. | Major depression not assessed |
| Georgiopoulos AM, Bryan TL, Wollan P, Yawn BP. Routine screening for postpartum depression. J Fam Pract. 2001;**50**:117. | Major depression not assessed |
| Gerardin P, Wendland J, Bodeau N, Galin A, Bialobos S, Tordjman S, et al. Depression during pregnancy: Is the developmental impact earlier in boys? A prospective case-control study. J Clin Psychiatry. 2011;**72**:378-87. | Sample selected for known distress, mental health diagnosis, or psychiatric setting |
| Gerardin P. Characteristics and clinical consequences of prenatal depression. Main results of a prospective case-control study on perinatal depression from pregnancy to one year-old infant. Neuropsychiatr Enfance eAdolesc. 2012;**60**:138-46. | Sample selected for known distress, mental health diagnosis, or psychiatric setting |
| Ghubash R, Abou-Saleh MT, Daradkeh TK. The validity of the Arabic Edinburgh Postnatal Depression Scale. Soc Psychiatry Psychiatr Epidemiol. 1997;**32**:474-6. | > 2 weeks between EPDS and diagnostic interview |
| Ghubash R, Abou-Saleh MT. Postpartum psychiatric illness in Arab culture: prevalence and psychosocial correlates. Br J Psychiatry. 1997;**171**:65-8. | > 2 weeks between EPDS and diagnostic interview |
| Ginsburg GS, Barlow A, Goklish N, Hastings R, Baker EV, Mullany B, et al. Postpartum depression prevention for reservation-based American Indians: Results from a Pilot Randomized Controlled Trial. Child Youth Care Forum. 2012;**41**:229-45. | Sample selected for known distress, mental health diagnosis, or psychiatric setting |
| Goeb JL, Férel S, Guetta J, Guibert J, Guedeney A, Coste J, et al. Assisted reproductive techniques when the Man is HIV Seropositive. Psychiatr Enfant. 2009;**52**:63-88. | Major depression not assessed |
| Goutaudier N, Lopez A, SéjournéN, Denis A, Chabrol H. Premature birth: subjective and psychological experiences in the first weeks following childbirth, a mixed-methods study. J Reprod Infant Psychol. 2011;**29**:364-73. | Major depression not assessed |
| Goyal D, Park VT, McNiesh S. Postpartum depression among Asian Indian mothers. MCN Am J Matern Child Nurs2015;**40**:256-61. | Major depression not assessed |
| Grant KA, Bautovich A, McMahon C, Reilly N, Leader L, Austin MP. Parental care and control during childhood: Associations with maternal perinatal mood disturbance and parenting stress. Arch Womens Ment Health. 2012;**15**:297-305. | Could not determine eligibility^a^ |
| Grant KA, McMahon C, Austin MP, Reilly N, Leader L, Ali S. Maternal prenatal anxiety, postnatal caregiving and infants' cortisol responses to the still-face procedure. Dev Psychobiol. 2009;**51**:625-37. | Could not determine eligibility^a^ |
| Grant KA, McMahon C, Reilly N, Austin MP. Maternal sensitivity moderates the impact of prenatal anxiety disorder on infant responses to the still-face procedure. Infant Behav Dev. 2010;**33**:453-62. | Could not determine eligibility^a^ |
| Grigoriadis S, de Camps Meschino D, Barrons E, Bradley L, Eady A, Fishell A, et al. Mood and anxiety disorders in a sample of Canadian perinatal women referred for psychiatric care. Arch Womens Ment Health. 2011;**14**:325-33. | Sample selected for known distress, mental health diagnosis, or psychiatric setting |
| Guedeney N, Fermanian J. Validation study of the French version of the Edinburgh Postnatal Depression Scale (EPDS): new results about use and psychometric properties. Eur Psychiatry. 1998;**13**:83-9. | Sample selected for known distress, mental health diagnosis, or psychiatric setting |
| Gutierrez-Zotes A, Labad J, Martin-Santos R, Garcia-Esteve L, Gelabert E, Jover M, et al. Coping strategies and postpartum depressive symptoms: A structural equation modelling approach. Eur Psychiatry. 2015;**30**:701-8. | Sample selected for known distress, mental health diagnosis, or psychiatric setting |
| Gutiérrez -Zotes JA, Farnós A, Vilella E, Labad J. Higher psychoticism as a predictor of thoughts of harming one's infant in postpartum women: a prospective study. Compr Psychiatry. 2013;**54**:1124-9. | Sample selected for known distress, mental health diagnosis, or psychiatric setting |
| Gutiérrez -Zotes A, Labad J, MartinSantos R, GarciaEsteve L, Gelabert E, Jover M, et al. Coping strategies and postpartum depressive symptoms: A structural equation modelling approach. Eur Psychiatry. 2015;**30**:701-8. | Sample selected for known distress, mental health diagnosis, or psychiatric setting |
| Hamdan A, Tamim H. Psychosocial risk and protective factors for postpartum depression in the United Arab Emirates. Arch Womens Ment Health. 2011;**14**:125-33. | Sample selected for known distress, mental health diagnosis, or psychiatric setting |
| Hamdan A, Tamim H. The relationship between postpartum depression and breastfeeding. Int J Psychiatry Med. 2012;**43**:243-59. | Sample selected for known distress, mental health diagnosis, or psychiatric setting |
| Hanusa BH, Scholle SH, Haskett RF, Spadaro K, Wisner KL. Screening for depression in the postpartum period: a comparison of three instruments. J Womens Health. 2008;**17**:585-96. | Sample selected for known distress, mental health diagnosis, or psychiatric setting |
| Harris B, Huckle P, Thomas R, Johns S, Fung H. The use of rating scales to identify post-natal depression. Br J Psychiatry. 1989;**154**:813-7. | Could not determine eligibility^a^ |
| Harris B, Othman S, Davies JA, Weppner GJ, Richards CJ, Newcombe RG, et al. Association between postpartum thyroid dysfunction and thyroid antibodies and depression. BMJ. 1992;**305**:152-6. | No validated interview to assess major depression |
| Harvey ST, Pun PK. Analysis of positive Edinburgh depression scale referrals to a consultation liaison psychiatry service in a two-year period. Int J Ment Health Nurs. 2007;**16**:161-7. | Sample selected for known distress, mental health diagnosis, or psychiatric setting |
| Hatton DC, HarrisonHohner J, Matarazzo J, Edwards P, Lewy A, Davis L. Missed antenatal depression among high risk women: A secondary analysis. Arch Womens Ment Health. 2007;**10**:121-3. | No validated interview to assess major depression |
| Henderson JJ, Evans SF, Straton JA, Priest SR, Hagan R. Impact of postnatal depression on breastfeeding duration. Birth. 2003;30(3):175-80. Erratum in: Birth. 2004 Mar;31(1):76. | Did not administer the SCID, CIDI, or MINI |
| Henshaw C, Foreman D, Cox J. Postnatal blues: a risk factor for postnatal depression. J Psychosom Obstet Gynecol. 2004;**25**:267-72. | Sample selected for known distress, mental health diagnosis, or psychiatric setting |
| Herz E, Thoma M, Umek W, Gruber K, Linzmayer L, Walcher W, et al. Non-psychotic post-partum depression. Geburtshilfe Frauenheilkd. 1997;**57**:282-8. | Major depression not assessed |
| Holden JM. Postnatal depression: its nature, effects, and identification using the Edinburgh Postnatal Depression scale. Birth. 1991;**18**:211-21. | No original data |
| Holt WJ. The detection of postnatal depression in general practice using the Edinburgh postnatal depression scale. N Z M J. 1995;**108**:57. | > 2 weeks between EPDS and diagnostic interview |
| Howard LM, Flach C, Mehay A, Sharp D, Tylee A. The prevalence of suicidal ideation identified by the Edinburgh Postnatal Depression Scale in postpartum women in primary care: findings from the RESPOND trial. BMC Pregnancy Childbirth. 2011;**11**:57-59. | Sample selected for known distress, mental health diagnosis, or psychiatric setting |
| Huang J, Zhang L, He M, Qiang X, Xiao X, Huang S, et al. Comprehensive evaluation of postpartum depression and correlations between postpartum depression and serum levels of homocysteine in Chinese women. Zhong Nan Da Xue Xue Bao Yi Xue BanZhong Nan Da Xue Xue Bao Yi Xue Ban. 2015;**40**:311-6. | No validated interview to assess major depression |
| Huang YC, Mathers NJ. Postnatal depression and the experience of South Asian marriage migrant women in Taiwan: survey and semi-structured interview study. Int J Nurs Stud. 2008;**45**:924-31. | Major depression not assessed |
| Husain N, Cruickshank K, Husain M, Khan S, Tomenson B, Rahman A. Social stress and depression during pregnancy and in the postnatal period in British Pakistani mothers: a cohort study. J Affect Disord. 2012;**140**:268-76. | Could not determine eligibility^a^ |
| Husain N, Kiran T, Sumra A, Naeem Zafar S, Ur Rahman R, Jafri F, et al. Detecting maternal depression in a low-income country: comparison of the self-reporting questionnaire and the Edinburgh Postnatal Depression Scale. J Trop Pediatr. 2014;**60**:129-33. | Could not determine eligibility^a^ |
| Ibanez G, Bernard JY, Rondet C, Peyre H, Forhan A, Kaminski M, et al. Effects of antenatal maternal depression and anxiety on children's early cognitive development: A prospective cohort study. PLOS ONE. 2015;**10**:Art e0135849. | Major depression not assessed |
| Ikeda M, Hayashi M, Kamibeppu K. The relationship between attachment style and postpartum depression. Attach Hum Dev. 2014;**16**:557-72. | > 2 weeks between EPDS and diagnostic interview |
| Inglis AJ, Hippman CL, Carrion PB, Honer WG, Austin JC. Mania and depression in the perinatal period among women with a history of major depressive disorders. Arch Womens Ment Health. 2014;**17**:137-43. | Sample selected for known distress, mental health diagnosis, or psychiatric setting |
| Jadresic E, Araya R, Jara C. Validation of the Edinburgh Postnatal Depression Scale (EPDS) in Chilean postpartum women. J Psychosom Obstet Gynecol. 1995;**16**:187-91. | No validated interview to assess major depression |
| Jaju S, Al Kharusi L, Gowri V. Antenatal prevalence of fear associated with childbirth and depressed mood in primigravid women. Indian J Psychiatry. 2015;**57**:158-61. | Sample selected for known distress, mental health diagnosis, or psychiatric setting |
| Jardri R, Maron M, Pelta J, Thomas P, Codaccioni X, Goudemand M, Delion P. Impact of midwives' training on postnatal depression screening in the first week post delivery: a quality improvement report. Midwifery. 2010;**26**:622-9. | > 2 weeks between EPDS and diagnostic interview |
| Ji S, Long Q, Newport DJ, Na H, Knight B, Zach EB, et al. Validity of depression rating scales during pregnancy and the postpartum period: impact of trimester and parity. J Psychiatr Res. 2011;**45**:213-9. | Sample selected for known distress, mental health diagnosis, or psychiatric setting |
| Josefsson A, Larsson C, Sydsjö G, Nylander PO. Temperament and character in women with postpartum depression. Arch Womens Ment Health. 2007;**10**:3-7. | Sample selected for known distress, mental health diagnosis, or psychiatric setting |
| Keshavarzi F, Yazdchi K, Rahimi M, Rezaei M, Farnia V, Davarinejad O, et al. Post partum depression and thyroid function. Iran J Psychiatry. 2011;**6**:117-20. | Major depression not assessed |
| Kirkan TS, Aydin N, Yazici E, Akcali Aslan P, Acemoglu H, Daloglu AG. The depression in women in pregnancy and postpartum period: A follow-up study. Int J Soc Psychiatry. 2015;**61**:343-9. | Sample selected for known distress, mental health diagnosis, or psychiatric setting |
| Klier CM, Muzik M, Dervic K, Mossaheb N, Benesch T, Ulm B, Zeller M. The role of estrogen and progesterone in depression after birth. J Psychiatr Res. 2007;**41**:273-9. | > 2 weeks between EPDS and diagnostic interview |
| Knorring LV. Book review of Depression in women with focus on the postpartum period. Nord J Psychiatry. 2003;**57**:390. | No validated interview to assess major depression |
| Kohlhoff J, Hickinbotham R, Knox C, Roach V, Barnett Am B. Antenatal psychosocial assessment and depression screening in a private hospital. Aust N Z J Obstet Gynaecol. 2016;**56**:173-8. | Major depression not assessed |
| Koss J, Bidzan M, Smutek J, Bidzan L. Influence of perinatal depression on labor-associated fear and mmotional attachment to the child in high-risk pregnancies and the first days after delivery. Med Sci Monit. 2016;**22**:1028-37. | Major depression not assessed |
| Lai BP, Tang AK, Lee DT, Yip AS, Chung TK. Detecting postnatal depression in Chinese men: a comparison of three instruments. Psychiatry Res. 2010;**180**:80-5. | No pregnant or postpartum women |
| Lau Y, Wang Y, Yin L, Chan KS, Guo X. Validation of the Mainland Chinese version of the Edinburgh Postnatal Depression Scale in Chengdu mothers. Int J Nurs Stud. 2010;**47**:1139-51. | Could not determine eligibility^a^ |
| Lawrie TA, Hofmeyr GJ, de Jager M, Berk M. Validation of the Edinburgh Postnatal Depression Scale on a cohort of South African women. S Afr Med J. 1998;**88**:1340-4. | No validated interview to assess major depression |
| Lee DT, Wong CK, Ungvari GS, Cheung LP, Haines CJ, Chung TK. Screening psychiatric morbidity after miscarriage: application of the 30-item General Health Questionnaire and the Edinburgh Postnatal Depression Scale. Psychosom Med. 1997;**59**:207-10. | No pregnant or postpartum women |
| Lee DT, Yip AS, Chan SS, Tsui MH, Wong WS, Chung TK. Postdelivery screening for postpartum depression. Psychosom Med. 2003;**65**:357-61. | Major depression not assessed |
| Lee DT, Yip AS, Chiu HF, Chung TK. Screening for postnatal depression using the double-test strategy. Psychosom Med. 2000;**62**:258-63. | Major depression not assessed |
| Lee DT, Yip AS, Chiu HF, Leung TY, Chung TK. Screening for postnatal depression: are specific instruments mandatory? J Affect Disord. 2001;**63**:233-8. | Major depression not assessed |
| Lee DT, Yip SK, Chiu HF, Leung TY, Chan KP, Chau IO, et al. Detecting postnatal depression in Chinese women. Validation of the Chinese version of the Edinburgh Postnatal Depression Scale. Br J Psychiatry. 1998;**172**:433-7. | No validated interview to assess major depression |
| Leverton TJ, Elliott SA. Is the EPDS a magic wand?: 1. A comparison of the Edinburgh Postnatal Depression Scale and health visitor report as predictors of diagnosis on the Present State Examination. J Reprod Infant Psychol. 2000;**18**:279-96. | Sample selected for known distress, mental health diagnosis, or psychiatric setting |
| Lewis BA, Gjerdingen DK, Avery MD, Guo H, Sirard JR, Bonikowske AR, Marcus BH. Examination of a telephone-based exercise intervention for the prevention of postpartum depression: design, methodology, and baseline data from The Healthy Mom study. Contemp Clin Trials. 2012;**33**:1150-8. | Major depression not assessed |
| Lewis BA, Gjerdingen DK, Avery MD, Sirard JR, Guo H, Schuver K, Marcus BH. A randomized trial examining a physical activity intervention for the prevention of postpartum depression: the healthy mom trial. Ment Health Phys Act. 2014;**7**:42-9. | Sample selected for known distress, mental health diagnosis, or psychiatric setting |
| Logsdon MC, Myers JA. Comparative performance of two depression screening instruments in adolescent mothers. J Womens Health. 2010;**19**:1123-8. | Not a sample of adults |
| Łukasik A, Błaszczyk K, Wojcieszyn M, Belowska A. Characteristic of affective disorders of the first week of puerperium. Ginekol Pol. 2003;**74**:1194-9. | No validated interview to assess major depression |
| Lundh W, Gyllang C. Use of the Edinburgh Postnatal Depression Scale in some Swedish child health care centres. Scand J Caring Sci. 1993;**7**:149-54. | No validated interview to assess major depression |
| Lydsdottir LB, Howard LM, Olafsdottir H, Thome M, Tyrfingsson P, Sigurdsson JF. The mental health characteristics of pregnant women with depressive symptoms identified by the Edinburgh Postnatal Depression Scale. J Clin Psychiatry. 2014;**75**:393-8. | > 2 weeks between EPDS and diagnostic interview |
| Mallett P, Andrew M, Hunter C, Smith J, Richards C, Othman S, et al. Cognitive function, thyroid status and postpartum depression. Acta Psychiatr Scand. 1995;**91**:243-6. | No validated interview to assess major depression |
| Maloney DM. Postnatal depression: a study of mothers in the metropolitan area of Perth, Western Australia. Aust J Midwifery. 1998;**11**:18-23. | Major depression not assessed |
| Mao HJ, Li HJ, Chiu H, Chan WC, Chen SL. Effectiveness of antenatal emotional self-management training program in prevention of postnatal depression in Chinese women. Perspect Psychiatr Care. 2012;**48**:218-24. | Sample selected for known distress, mental health diagnosis, or psychiatric setting |
| Martin-Santos R, Gelabert E, Subira S, Gutierrezzotes A, Langorh K, Jover M, et al. Research Letter: Is neuroticism a risk factor for postpartum depression? Psychol Med. 2012;**42**:1559-65. | No original data |
| Mason L, Poole H. Healthcare professionals' views of screening for postnatal depression. Community Pract. 2008;**81**:30-4. | No pregnant or postpartum women |
| Matijasevich A, Munhoz TN, Tavares BF, Barbosa AP, da Silva DM, Abitante MS, et al. Validation of the Edinburgh Postnatal Depression Scale (EPDS) for screening of major depressive episode among adults from the general population. BMC Psychiatry. 2014;**14**:284. | No pregnant or postpartum women |
| Matthey S, Barnett B, Howie P, Kavanagh DJ. Diagnosing postpartum depression in mothers and fathers: whatever happened to anxiety? J Affect Disord. 2003;74(2):139-47. | Did not administer the SCID, CIDI, or MINI |
| Matthey S, Barnett BEW, Elliott A. Vietnamese and Arabic women's responses to the Diagnostic Interview Schedule (depression) and self-report questionnaires: cause for concern. Aust N Z J Psychiatry. 1997;31(3):360-9. | Did not administer the SCID, CIDI, or MINI |
| Matthey S, Barnett B, Kavanagh DJ, Howie P. Validation of the Edinburgh Postnatal Depression Scale for men, and comparison of item endorsement with their partners. J Affect Disord. 2001;**64**:175-84. | Did not administer the SCID, CIDI, or MINI |
| Matthey S, Valenti B, Souter K, Ross-Hamid C. Comparison of four self-report measures and a generic mood question to screen for anxiety during pregnancy in English-speaking women J Affect Disord. 2013;**148**:347-51. | Sample selected for known distress, mental health diagnosis, or psychiatric setting |
| Matthey S. Differentiating between Transient and Enduring distress on the Edinburgh Depression Scale within screening contexts. J Affect Disord. 2016;**196**:252-58. | Sample selected for known distress, mental health diagnosis, or psychiatric setting |
| Matthey S. Using the Edinburgh Postnatal Depression Scale to screen for anxiety disorders. Depress Anxiety. 2008;**25**:926-31. | No pregnant or postpartum women |
| Mauri M, Banti S, Borri C, Rambelli C, Ramacciotti D, Oppo A, et al. Depressive Symptomatology in Pregnancy Detected with EPDS: the Problem of False Positive. Eur Psychiatry. 2010;**25**:1403. | Sample selected for known distress, mental health diagnosis, or psychiatric setting |
| Mazhari S, Nakhaee N. Validation of the Edinburgh Postnatal Depression Scale in an Iranian sample. Arch Womens Ment Health. 2007;**10**:293-7. | No validated interview to assess major depression |
| Mazzeo SE, SlofOp't Landt MC, Jones I, Mitchell K, Kendler KS, Neale MC, et al. Associations among postpartum depression, eating disorders, and perfectionism in a population-based sample of adult women. Int J Eat Disord. 2006;**39**:202-11. | Major depression not assessed |
| McMahon CA, Boivin J, Gibson FL, Hammarberg K, Wynter K, Fisher JR. Older maternal age and major depressive episodes in the first two years after birth: Findings from the Parental Age and Transition to Parenthood Australia (PATPA) study. J Affect Disord. 2015;**175**:454-62. | Major depression not assessed |
| Meltzer-Brody S, Zerwas S, Leserman J, Von Holle A, Regis T, Bulik C. Eating disorders and trauma history in women with perinatal depression. J Womens Health. 2011;**20**:863-70. | Sample selected for known distress, mental health diagnosis, or psychiatric setting |
| Meuti V, Aceti F, Giacchetti N, Carluccio GM, Zaccagni M, Marini I, et al. Perinatal depression and patterns of attachment: a critical risk factor? Depress Res Treat. 2015;**2015**:105012. | Sample selected for known distress, mental health diagnosis, or psychiatric setting |
| Milgrom J, Gemmill AW, Ericksen J, Burrows G, Buist A, Reece J. Treatment of postnatal depression with cognitive behavioural therapy, sertraline and combination therapy: A randomised controlled trial. Aust N Z J Psychiatry. 2015;**49**:236-245. | Sample selected for known distress, mental health diagnosis, or psychiatric setting |
| Miller L, Gur M, Shanok A, Weissman M. Interpersonal psychotherapy with pregnant adolescents: two pilot studies. J Child Psychol Psychiatry. 2008;**49**:733-42. | Not a sample of adults |
| Moayedoddin A, Moser D, Nanzer N. The impact of brief psychotherapy centred on parenthood on the anxio-depressive symptoms of mothers during the perinatal period. Swiss Med Wkly. 2013;**143**:w13769. | Sample selected for known distress, mental health diagnosis, or psychiatric setting |
| Murray D, Cox JL, Chapman G, Jones P. Childbirth: life event or start of a long-term difficulty? Further data from the Stoke-on-Trent controlled study of postnatal depression. Br J Psychiatry. 1995;**166**:595-600. | No validated interview to assess major depression |
| Murray D, Cox JL. Screening for depression during pregnancy with the Edinburgh Depression Scale (EPDS). J Reprod Infant Psychol. 1990;**8**:99-107. | No validated interview to assess major depression |
| Murray L, Carothers AD. The validation of the Edinburgh Post-natal Depression Scale on a community sample. Br J Psychiatry. 1990;**157**:288-90. | No validated interview to assess major depression |
| O'Brien LM, Heycock EG, Hanna M, Jones PW, Cox JL. Postnatal depression and faltering growth: A community study. Pediatrics. 2004;**113**:1242-7. | Did not administer the SCID, CIDI, or MINI |
| O'Mahen H, Himle JA, Fedock G, Henshaw E, Flynn H. A pilot randomized controlled trial of cognitive behavioral therapy for perinatal depression adapted for women with low incomes. Depress Anxiety. 2013;**30**:679-87. | Sample selected for known distress, mental health diagnosis, or psychiatric setting |
| O'Neill T. Postnatal depression--aetiological factors. Ir Med J. 1990;**83**:17-18. | > 2 weeks between EPDS and diagnostic interview |
| Ortiz Collado MA, Saez M, Favrod J, Hatem M. Antenatal psychosomatic programming to reduce postpartum depression risk and improve childbirth outcomes: a randomized controlled trial in Spain and France. BMC Pregnancy & Childbirth. 2014;**14**:22. | Major depression not assessed |
| Owoeye AO, Aina OF, Morakinyo O. Risk factors of postpartum depression and EPDS scores in a group of Nigerian women. Trop Doct. 2006;**36**:100-3. | Sample selected for known distress, mental health diagnosis, or psychiatric setting |
| Parker G, Hegarty B, Granville-Smith I, Ho J, Paterson A, Gokiert A, Hadzi-Pavlovic D. Is essential fatty acid status in late pregnancy predictive of post-natal depression?. Acta Psychiatr Scand. 2015;**131**:148-56. | Sample selected for known distress, mental health diagnosis, or psychiatric setting |
| Parker GB, Hegarty B, Paterson A, Hadzi-Pavlovic D, Granville-Smith I, Gokiert A. Predictors of post-natal depression are shaped distinctly by the measure of 'depression'. J Affect Disord. 2015;**173**:239-44. | Sample selected for known distress, mental health diagnosis, or psychiatric setting |
| Pawlby S, Sharp D, Hay D, O'Keane V. Postnatal depression and child outcome at 11 years: the importance of accurate diagnosis. J Affect Disord. 2008;**107**:241-5. | Did not administer the SCID, CIDI, or MINI |
| Patton GC, Romaniuk H, Spry E, Coffey C, Olsson C, Doyle LW, et al. Prediction of perinatal depression from adolescence and before conception (VIHCS): 20-year prospective cohort study. Lancet. 2015;**386**:875-83. | Major depression not assessed |
| Peindl KS, Wisner KL, Hanusa BH. Identifying depression in the first postpartum year: guidelines for office-based screening and referral. J Affect Disord. 2004;**80**:37-44. | Sample selected for known distress, mental health diagnosis, or psychiatric setting |
| Phillips J, Sharpe L, Nemeth D. Maternal psychopathology and outcomes of a residential mother-infant intervention for unsettled infant behaviour. Aust N Z J Psychiatry. 2010;**44**:280-9. | > 2 weeks between EPDS and diagnostic interview |
| Piacentini D, Leveni D, Primerano G, Cattaneo M, Volpi L, Biffi G, Mirabella F. Prevalence and risk factors of postnatal depression among women attending antenatal courses. Epidemiologia Psichiatr Soc. 2009;**18**:214-20. | > 2 weeks between EPDS and diagnostic interview |
| Pitanupong J, Liabsuetrakul T, Vittayanont A. Validation of the Thai Edinburgh Postnatal Depression Scale for screening postpartum depression. Psychiatry Res. 2007;**149**:253-9. | No validated interview to assess major depression |
| Pollock JI, Manaseki-Holland S, Patel V. Detection of depression in women of child-bearing age in non-Western cultures: a comparison of the Edinburgh Postnatal Depression Scale and the Self-Reporting Questionnaire-20 in Mongolia. J Affect Disord. 2006;**92**:267-71. | Not a sample of adults |
| Priest SR, Henderson J, Evans SF, Hagan R. Stress debriefing after childbirth: a randomised controlled trial. Med J Aust. 2003;**178**:542-5. | Did not administer the SCID, CIDI, or MINI |
| Quispel C, Schneider TA, Hoogendijk WJ, Bonsel GJ, Lambregtse-van den Berg MP. Successful five-item triage for the broad spectrum of mental disorders in pregnancy–a validation study. BMC Pregnancy Childbirth. 2015;**15**:51. | No major depression cases |
| Reck C, Stehle E, Reinig K, Mundt C. Maternity blues as a predictor of DSM-IV depression and anxiety disorders in the first three months postpartum. J Affect Disord. 2009;**113**:77-87. | Sample selected for known distress, mental health diagnosis, or psychiatric setting |
| Reck C, Struben K, Backenstrass M, Stefenelli U, Reinig K, Fuchs T, et al. Prevalence, onset and comorbidity of postpartum anxiety and depressive disorders. Acta Psychiatr Scand. 2008;**118**:459-68. | > 2 weeks between EPDS and diagnostic interview |
| Regmi S, Sligl W, Carter D, Grut W, Seear M. A controlled study of postpartum depression among Nepalese women: validation of the Edinburgh Postpartum Depression Scale in Kathmandu. Trop Med Int Health. 2002;**7**:378-82. | Major depression not assessed |
| Robakis TK, Williams KE, Crowe S, Kenna H, Gannon J, Rasgon NL. Optimistic outlook regarding maternity protects against depressive symptoms postpartum. Arch Womens Ment Health. 2015;**18**:197-208. | No validated interview to assess major depression |
| Roca A, Imaz ML, Torres A, Plaza A, Subira S, Valdes M, et al. Unplanned pregnancy and discontinuation of SSRIs in pregnant women with previously treated affective disorder. J Affect Disord. 2013;**150**:807-13. | Sample selected for known distress, mental health diagnosis, or psychiatric setting |
| Rojas G, Fritsch R, Solis J, Gonzalez M, Guajardo V, Araya R. Quality of life of women depressed in the post-partum period. Rev Med Chil. 2006;**134**:713-20. | Sample selected for known distress, mental health diagnosis, or psychiatric setting |
| Rubertsson C, Borjesson K, Berglund A, Josefsson A, Sydsjo G. The Swedish validation of Edinburgh Postnatal Depression Scale (EPDS) during pregnancy. Nord J Psychiatry. 2011;**65**:414-8. | No validated interview to assess major depression |
| Saleh ES, El-Bahei W, El-Hadidy MA, Zayed A. Predictors of postpartum depression in a sample of Egyptian women. Neuropsychiatr Dis Treat. 2012;**9**:15-24. | EPDS not administered |
| Sanjuan J, MartinSantos R, GarciaEsteve L, Carot JM, Guillamat R, GutierrezZotes A, et al. Mood changes after delivery: Role of the serotonin transporter gene. Br J Psychiatry. 2008;**193**:383-8. | Sample selected for known distress, mental health diagnosis, or psychiatric setting |
| Santos IS, Matijasevich A, Tavares BF, Barros AJ, Botelho IP, Lapolli C, et al. Validation of the Edinburgh Postnatal Depression Scale (EPDS) in a sample of mothers from the 2004 Pelotas Birth Cohort Study.Cad Saude Publica. 2007;**23**:2577-88. | No validated interview to assess major depression |
| Santos IS, Matijasevich A, Tavares BF, da Cruz Lima AC, Riegel RE, Lopes BC. Comparing validity of Edinburgh scale and SRQ20 in screening for post-partum depression. Clin Pract Epidemiol Ment Health. 2007;**3**:18. | No validated interview to assess major depression |
| Savarimuthu RJ, Ezhilarasu P, Charles H, Antonisamy B, Kurian S, Jacob KS. Post-partum depression in the community: a qualitative study from rural South India. Int J Soc Psychiatry. 2010;**56**:94-102. | Could not determine eligibility^a^ |
| Séjourné N, Alba J, Onorrus M, Goutaudier N, Chabrol H. Intergenerational transmission of postpartum depression. J Reprod Infant Psychol. 2011;**29**:115-24. | No validated interview to assess major depression |
| Seth S, Lewis AJ, Saffery R, Lappas M, Galbally M. Maternal prenatal mental health and placental 11 beta-HSD2 gene expression: initial findings from the Mercy Pregnancy and Emotional Wellbeing study. Int J Mol Sci. 2015;**16**:27482-96. | Major depression not assessed |
| Simpson W, Glazer M, Michalski N, Steiner M, Frey BN. Comparative efficacy of the generalized anxiety disorder 7-item scale and the Edinburgh Postnatal Depression Scale as screening tools for generalized anxiety disorder in pregnancy and the postpartum period. Can J Psychiatry. 2014;**59**:434-40. | Sample selected for known distress, mental health diagnosis, or psychiatric setting |
| Sit DK, Flint C, Svidergol D, White J, Wimer M, Bish B, Wisner KL. Best practices: an emerging best practice model for perinatal depression care. Psychiatr Serv. 2009;**60**:1429-31. | No validated interview to assess major depression |
| Slade P, Morrell CJ, Rigby A, Ricci K, Spittlehouse J, Brugha TS. Postnatal women's experiences of management of depressive symptoms: a qualitative study. Br J Gen Pract. 2010;**60**:e440-e448. | Major depression not assessed |
| Smith-Nielsen J, Steele H, Mehlhase H, Cordes K, Steele M, Harder S, Vaever MS. Links among high EPDS scores, state of mind regarding attachment, and symptoms of personality disorder. J Pers Disord. 2015;**29**:771-93. | Sample selected for known distress, mental health diagnosis, or psychiatric setting |
| Sundaram S, Harman JS, Cook RL. Maternal morbidities and postpartum depression: An analysis using the 2007 and 2008 pregnancy risk assessment monitoring system. Womens Health Issues. 2014;**24**:e381-8. | EPDS not administered |
| Sutter-Dallay AL, Giaconne-Marcesche V, Glatigny-Dallay E, Verdoux H. Women with anxiety disorders during pregnancy are at increased risk of intense postnatal depressive symptoms: a prospective survey of the MATQUID cohort. Eur Psychiatry. 2004;**19**:459-63. | > 2 weeks between EPDS and diagnostic interview |
| Tam LW, Newton RP, Dern M, Parry BL. Screening women for postpartum depression at well baby visits: resistance encountered and recommendations. Arch Womens Ment Health. 2002;**5**:79-82. | Sample selected for known distress, mental health diagnosis, or psychiatric setting |
| Tan EC, Chua TE, Lee TMY, Tan HS, Ting JLY, Chen HY. Case-control study of glucocorticoid receptor and corticotrophin-releasing hormone receptor gene variants and risk of perinatal depression. BMC Pregnancy Childbirth. 2015;**15**:283. | Major depression not assessed |
| Tang Y, Shi S, Lu W, Chen Y, Wang Q, Zhu Y, et al. Prenatal psychological prevention trial on postpartum anxiety and depression. Chin Ment Health J. 2009;**23**:83-89. | Could not determine eligibility^a^ |
| Teng HW, Hsu CS, Shih SM, Lu ML, Pan JJ, Shen WW. Screening postpartum depression with the Taiwanese version of the Edinburgh Postnatal Depression scale. Compr Psychiatry. 2005;**46**:261-65. | Could not determine eligibility^a^ |
| Tesfaye M, Hanlon C, Wondimagegn D, Alem A. Detecting postnatal common mental disorders in Addis Ababa,​ Ethiopia: validation of the Edinburgh Postnatal Depression Scale and Kessler Scales. J Affect Disord. 2010;**122**:102-8. | No validated interview to assess major depression |
| Tharner A, Luijk MPCM, van IJzendoorn MH, BakermansKranenburg MJ, Jaddoe VWV, Hofman A, et al. Maternal lifetime history of depression and depressive symptoms in the prenatal and early postnatal period do not predict infant-mother attachment quality in a large,​ population-based Dutch cohort study. Attach Hum Dev. 2012;**14**:63-81. | > 2 weeks between EPDS and diagnostic interview |
| Thorpe K. A study of the use of the Edinburgh Postnatal Depression Scale with parent groups outside the postpartum period. J Reprod Infant Psychol. 1993;**11**:119-25. | No pregnant or postpartum women |
| Tietz A, Zietlow AL, Reck C. Maternal bonding in mothers with postpartum anxiety disorder: the crucial role of subclinical depressive symptoms and maternal avoidance behaviour. Arch Womens Ment Health. 2014;**17**:433-42. | Sample selected for known distress, mental health diagnosis, or psychiatric setting |
| Tissot H, Favez N, Frascarolo-Moutinot F, Despland JN. Assessing postpartum depression: Evidences for the need of multiple methods. Eur Rev App Psychol. 2015**;65**:61-6. | Did not administer the SCID, CIDI, or MINI |
| Ueda M, Yamashita H, Yoshida K. Impact of infant health problems on postnatal depression: pilot study to evaluate a health visiting system. Psychiatry Clin Neurosci. 2006;**60**:182-9. | > 2 weeks between EPDS and diagnostic interview |
| Uguz F, Akman C, Sahingoz M, Kaya N, Kucur R. One year follow-up of post-partum-onset depression: the role of depressive symptom severity and personality disorders. J Psychosom Obstet Gynecol. 2009;**30**:141-5. | Sample selected for known distress, mental health diagnosis, or psychiatric setting |
| Uwakwe R, Okonkwo JE. Affective (depressive) morbidity in puerperal Nigerian women: validation of the Edinburgh Postnatal Depression Scale. Acta Psychiatr Scand. 2003;**107**:251-9. | No validated interview to assess major depression |
| Venkatesh KK, Zlotnick C, Triche EW, Ware C, Phipps MG. Accuracy of brief screening tools for identifying postpartum depression among adolescent mothers. Pediatrics. 2014;**133**:e45-e45. | Not a sample of adults |
| Venter MD, Smets J, Raes F, Wouters K, Franck E, Hanssens M, et al. Impact of childhood trauma on postpartum depression: A prospective study. Arch Womens Ment Health. 2016;**19**:337-42. | Major depression not assessed |
| Verkerk GJ, Denollet J, Van Heck GL, Van Son MJ, Pop VJ. Personality factors as determinants of depression in postpartum women: a prospective 1-year follow-up study. Psychosom Med. 2005;**67**:632-7. | No validated interview to assess major depression |
| Verkerk GJM, Pop VJM, Van Son MJM, Van Heck GL. Prediction of depression in the postpartum period: A longitudinal follow-up study in high-risk and low-risk women. J Affect Disord. 2003;**77**:159-66. | > 2 weeks between EPDS and diagnostic interview |
| Viktorin A, Meltzer-Brody S, Kuja-Halkola R, Sullivan PF, Landen M, Lichtenstein P, Magnusson PK. Heritability of perinatal depression and genetic overlap with nonperinatal depression. Am J Psychiatry. 2016;**173**:158-65. | EPDS not administered |
| Wang Y, Guo X, Lau Y, Chan KS, Yin L, Chen J. Psychometric evaluation of the Mainland Chinese version of the Edinburgh Postnatal Depression Scale. Int J Nurs Stud. 2009;**46**:813-23. | Could not determine eligibility^a^ |
| Warner R, Appleby L, Whitton A, Faragher B. Attitudes toward motherhood in postnatal depression: development of the Maternal Attitudes Questionnaire. J Psychosom Res. 1997;**43**:351-8. | Sample selected for known distress, mental health diagnosis, or psychiatric setting |
| Warnock FF, Bakeman R, Shearer K, Misri S, Oberlander T. Caregiving behavior and interactions of prenatally depressed mothers (antidepressant-treated and non-antidepressant-treated) during newborn acute pain. Infant Ment Health J. 2009;**30**:384-406. | Could not determine eligibility^a^ |
| Weobong B, Akpalu B, Doku V, Agyei SO, Hurt L, Kirkwood B, Prince M. The comparative validity of screening scales for postnatal common mental disorder in Kintampo, Ghana. J Affect Disord. 2009;**113**:109-17. | No validated interview to assess major depression |
| Werrett J, Clifford C. Validation of the Punjabi version of the Edinburgh postnatal depression scale (EPDS). Int J Nurs Stud. 2006;**43**:227-36. | Major depression not assessed |
| Wickberg B, Hwang CP. Counselling of postnatal depression: a controlled study on a population based Swedish sample. J Affect Disord. 1996;**39**:209-16. | No validated interview to assess major depression |
| Wickberg B, Hwang CP. The Edinburgh Postnatal Depression Scale: validation on a Swedish community sample. Acta Psychiatr Scand. 1996;**94**:181-84. | No validated interview to assess major depression |
| Wu M, Li X, Feng B, Wu H, Qiu C, Zhang W. Correlation between sleep quality of third-trimester pregnancy and postpartum depression. Med Sci Monit. 2014;**20**:2740-5. | Could not determine eligibility^a^ |
| Yamashita H, Yoshida K, Nakano H, Tashiro N. Postnatal depression in Japanese women. Detecting the early onset of postnatal depression by closely monitoring the postpartum mood. J Affect Disord. 2000;**58**:145-54. | No validated interview to assess major depression |
| Yonkers KA, Ramin SM, Rush AJ, Navarrete CA, Carmody T, March D, et al. Onset and persistence of postpartum depression in an inner-city maternal health clinic system. Am J Psychiatry. 2001;**158**:1856-63. | Sample selected for known distress, mental health diagnosis, or psychiatric setting |
| Yoshida K, Yamashita H, Ueda M, Tashiro N. Postnatal depression in Japanese mothers and the reconsideration of 'Satogaeri bunben'. Pediatr Int. 2001;**43**:189-93. | No validated interview to assess major depression |
| Zammit S, Thomas K, Thompson A, Horwood J, Menezes P, Gunnell D, et al. Maternal tobacco, cannabis and alcohol use during pregnancy and risk of adolescent psychotic symptoms in offspring. Br J Psychiatry. 2009;**195**:294-300. | No pregnant or postpartum women |
| Zelkowitz P, Milet TH. Postpartum psychiatric disorders: Their relationship to psychological adjustment and marital satisfaction in the spouses. J Abnorm Psychol. 1996;**105**:281-5. | Sample selected for known distress, mental health diagnosis, or psychiatric setting |
| Zlotnick C, Capezza NM, Parker D. An interpersonally based intervention for low-income pregnant women with intimate partner violence: A pilot study. Arch Womens Ment Health. 2011;**14**:55-65. | Sample selected for known distress, mental health diagnosis, or psychiatric setting |
| Zubaran C, Foresti K, Schumacher MV, Amoretti AL, Thorell MR, Muller LC. The correlation between postpartum depression and health status. Matern & Child Health J. 2010;**14**:751-7. | > 2 weeks between EPDS and diagnostic interview |

**^a^**It was not possible to determine eligibility based on published report, and we were not able to obtain clarification from authors despite multiple attempts

**Supplementary Table 2a. Characteristics of Included Primary Studies (N = 46)**

| **First Author, Year** | **Country** | **Recruited Population** | **Classification System** | **Total N** | **N (%) Major Depression** |  |  |  |
| --- | --- | --- | --- | --- | --- | --- | --- | --- |
| **Structured Clinical Interview for DSM Disorders** | | | | | |  |  |  |
| **Aceti, 2012^1^** | Italy | Pregnant women in the third trimester | DSM-IV | 44 | 22 (50) |  |  |  |
| **Barnes, 2009^2^** | UK | Socially disadvantaged mothers at 2 months postpartum | DSM-III-R | 347 | 25 (7) |  |  |  |
| **Bavle, 2016^3^** | India | Pregnant women recruited from an outpatient obstetrics department in a tertiary care hospital | DSM-IV | 318 | 6 (2) |  |  |  |
| **Beck, 2001^4^** | USA | Postpartum mothers | DSM-IV | 150 | 18 (12) |  |  |  |
| **Bunevicius, 2009^5^** | Lithuania | Pregnant women 12 to 16 weeks pregnant attending an obstetric clinic | DSM-III-R | 230 | 12 (5) |  |  |  |
| **Chaudron, 2010^6^** | USA | Postpartum women recruited from Well-Child Care visits with infants 0-14 months of age | DSM-IV | 187 | 70 (37) |  |  |  |
| **de Figueiredo, 2015^7^** | Brazil | Postpartum women enrolled in prenatal care outpatient services in a Brazilian city | DSM-IV | 241 | 94 (39) |  |  |  |
| **Garcia-Esteve, 2003^8^** | Spain | Women at 6 weeks postpartum | DSM-III-R | 334 | 36 (11) |  |  |  |
| **Giardinelli, 2012^9^** | Italy | Women between 28 and 32 weeks pregnant recruited from a obstetric course in Florence | DSM-IV | 588 | 28 (5) |  |  |  |
| **Helle, 2015^10^** | Germany | Mothers with very low birthweight and normal weigh infants between 4 and 6 weeks postpartum | DSM-IV | 224 | 12 (5) |  |  |  |
| **Hickey, 1997^11^** | Australia | Postpartum women recruited in the hospital after delivery | DSM-III-R | 72 | 31 (43) |  |  |  |
| **Howard, 2018^12^** | UK | Pregnant women recruited from an inner-city London maternity service | DSM-IV | 527 | 130 (25) |  |  |  |
| **Leonardou, 2009^13^** | Greece | Postpartum women recruited from private and public maternity wards on their second day postpartum | DSM-III-R | 81 | 4 (5) |  |  |  |
| **Navarro, 2007^14^** | Spain | Women presenting for postpartum care at 6 weeks | DSM-IV | 401 | 84 (21) |  |  |  |
| **Nakić Radoš, 2013^15^** | Croatia | Women between 6 and 8 weeks postpartum | DSM-IV-TR | 272 | 10 (4) |  |  |  |
| **Phillips, 2009^16^** | Australia | Postpartum mothers with unsettled infants | DSM-IV | 158 | 42 (27) |  |  |  |
| **Prenoveau, 2013^17^** | UK | Postpartum women at 10 months recruited from mixed health centres. | DSM-IV | 219 | 20 (9) |  |  |  |
| **Robertson-Blackmore, 2013^18^** | USA | Women at 18 weeks gestation | DSM-IV-TR | 358 | 29 (8) |  |  |  |
| **Rochat, 2013^19^** | South Africa | Women recruited from their antenatal appointment at a primary health care clinic between 26 and 34 weeks of pregnancy | DSM-IV | 104 | 50 (48) |  |  |  |
| **Siu, 2012^20^** | China | Postpartum women | DSM-IV | 805 | 126 (16) |  |  |  |
| **Stewart, 2013^21^** | Malawi | Pregnant women attending an antenatal clinic in rural Malawi | DSM-IV | 186 | 34 (18) |  |  |  |
| **Tandon, 2012^22^** | USA | Pregnant and postpartum women enrolled in home visitation programs | DSM IV | 89 | 25 (28) |  |  |  |
| **Tendais, 2014^23^** | Portugal | Pregnant women recruited in an obstetrics outpatient unit | DSM-IV | 141 | 18 (13) |  |  |  |
| **Töreki, 2013^24^** | Hungary | Women at 12 weeks antenatal | DSM-IV | 219 | 7 (3) |  |  |  |
| **Töreki, 2014^25^** | Hungary | Women between 6 and 8 weeks postpartum | DSM-IV | 265 | 8 (3) |  |  |  |
| **Tran, 2011^26^** | Vietnam | Pregnant and postpartum Vietnamese women recruited from the commune health centre | DSM-IV | 359 | 52 (14) |  |  |  |
| **Turner, 2009^27^** | Italy | Women from a regional epilepsy center in Italy between 5 and 8 weeks postpartum | DSM-IV-TR | 54 | 5 (9) |  |  |  |
| **Vega-Dienstmaier, 2002^28^** | Peru | Women up to 12 months postpartum | DSM-IV | 306 | 19 (6) |  |  |  |
| **Composite International Diagnostic Interview** | | | | | |  |  |  |
| **Fisher, 2010^29^** | Australia | Postpartum women recruited in Australian maternal and child health centres at 6 months postpartum | DSM-IV | 192 | 1 (1) |  |  |  |
| **Rowe, 2008^30^** | Australia | English speaking women admitted with their up to 1-year-old infants to private parenting centers | DSM-IV | 122 | 23 (19) |  |  |  |
| **Yonkers, 2014^31^** | USA | Women at 17 weeks gestation | DSM-IV | 2634 | 170 (6) |  |  |  |
| **Mini Neurospsychiatric Diagnostic Interview** | | | | | |  |  |  |
| **Alvarado, 2015^32^** | Chile | Pregnant women up to 28 weeks gestation | DSM-IV | 111 | 38 (34) |  |  |  |
| **Alvarado-Esquivel, 2006^33^** | Mexico | Women within 3 months postpartum | DSM-IV | 91 | 10 (11) |  |  |  |
| **Alvarado-Esquivel, 2016^34^** | Mexico | Pregnant women recruited at a public hospital in Durango City, Mexico | DSM-IV | 184 | 12 (7) |  |  |  |
| **Bakare, 2014^35^** | Nigeria | Postpartum women | DSM-IV | 405 | 62 (15) |  |  |  |
| **Couto, 2015^36^** | Brazil | Women in their second trimester of pregnancy recruited at antenatal care in a public hospital | DSM-IV-TR | 173 | 36 (21) |  |  |  |
| **Comasco, 2016^37^** | Sweden | Pregnant women | DSM-IV | 220 | 18 (8) |  |  |  |
| **Eapen, 2013^38^** | Australia | Women attending an antenatal clinic in Sydney | DSM-IV | 131 | 26 (20) |  |  |  |
| **Fernandes, 2011^39^** | India | Rural women in their third trimester | DSM-IV | 133 | 27 (20) |  |  |  |
| **Figueira, 2009^40^** | Brazil | Postpartum mothers recruited from hospitalization records | DSM-IV | 239 | 18 (8) |  |  |  |
| **Imbula, 2012^41^** | Democratic Republic of Congo | Women between 1 and 10 months postpartum recruited from 'well-baby' clinics | DSM-IV-TR | 117 | 29 (25) |  |  |  |
| **Khalifa, 2015^42^** | Sudan | Women at 3 months postpartum | ICD-10 | 40 | 18 (45) |  |  |  |
| **Roomruangwong, 2016^43^** | Thailand | Pregnant women at the end of their term | DSM-IV-TR | 126 | 1 (1) |  |  |  |
| **Su, 2007^44^** | Taiwan | Women in their second and third trimesters | DSM-IV | 185 | 23 (12) |  |  |  |
| **Thiagayson, 2013^45^** | Singapore | Inpatient high-risk pregnant women at 23 or more weeks of gestation | DSM-IV | 200 | 22 (11) |  |  |  |
| **Usuda, 2016^46^** | Japan | Pregnant women between 12 and 24 weeks of gestation recruited at maternity hospital in Japan | DSM-IV | 177 | 2 (1) |  |  |  |

**Abbreviations**: DSM: Diagnostic and Statistical Manual of Mental Disorders; ICD: International Classification of Diseases; UK: United Kingdom; USA: United States of America.

**Supplementary Table 2b. Characteristics of Eligible Primary Studies That Did Not Provide Data for the Present Study (N = 19)**

| **First Author, Year** | **Country** | **Recruited Population** | **Total N** | **N (%) Major Depression** |
| --- | --- | --- | --- | --- |
| **Structured Clinical Interview for DSM Disorders** | | | | |
| **Aydin, 2004^47^** | Turkey | Women within their first postpartum year attending primary health care clinics in the province of Erzurum | 341 | 34 (10) |
| **Banti, 2011^48^** | Italy | Pregnant women presenting to the local health service in the region of Tuscany between 12 and 15 weeks gestation | 1066 | NR |
| **Brodey, 2016^49^** | USA | Pregnant women recruited from private obstetrics clinics in Atlanta, Georgia and Tulsa, Oklahoma as well as women within 150 days postpartum | 879 | NR |
| **Chibanda, 2010^50^** | Zimbabwe | HIV-infected and uninfected women attending two primary care clinics in Chitungwiza six weeks postpartum | 210 | NR |
| **Gausia, 2007^51^** | Bangladesh | Women 6 to 8 weeks postpartum attending an urban childhood immunization clinic in Bangladesh | 100 | 3 (3) |
| **Gorman, 2004^52^** | France, Ireland, Italy, USA, UK, Portugal, Austria, Switzerland | Women in their the third trimester of pregnancy and at 6 months postpartum from 10 sites in 8 countries | 289 | 10 (4) |
| **Li, 2011^53^** | China | Women between 2 and 12 weeks postpartum recruited from postnatal clinics of the three regional public hospitals in Changsha, China | 387 | 24 (6) |
| **Moses-Kolko, 2012^54^** | USA | Postpartum women within 16 weeks of delivery | 33 | 13 (39) |
| **Stuebe, 2013^55^** | USA | Women in the third trimester of a singleton pregnancy who intended to breastfeed for at least 3 months | 47 | 8 (17) |
| **Composite International Diagnostic Interview** | | | | |
| **Bergink, 2011^56^** | The Netherlands | Pregnant women at 12 weeks gestation from 5 community midwifery practices in and around the city of Eindhoven | 845 | 47 (6) |
| **Mahmud, 2003^57^** | Malaysia | Women between 4 and 12 weeks postpartum attending a health clinic in Kedah | 64 | 9 (14) |
| **Mini Neurospsychiatric Diagnostic Interview** | | | | |
| **Adewuya, 2006^58^** | Nigeria | Women between 32 and 36 weeks pregnant recruited from the antenatal clinics in western Nigeria | 86 | 9 (10) |
| **Adouard, 2005^59^** | France | Women between 28 and 34 weeks gestation attending antenatal consultations for pregnancy complication in a major Parisian maternity facility | 60 | 15 (25) |
| **Agoub, 2005^60^** | Morocco | Postpartum women at their first postnatal visit 15 to 20 days after delivery | 144 | 27 (19) |
| **Benvenuti, 1999^61^** | Italy | Women between 8 and 12 weeks postpartum in Florence’s metropolitan area | 113 | 18 (16) |
| **Berle, 2003^62^** | Norway | Women attending routine postnatal visits between 6 and 12 weeks postpartum | 100 | 27 (27) |
| **Christl, 2013^63^** | Australia | Mothers with unsettled infants aged up to 12 months from a family care centre in Canterbury | 232 | 13 (6) |
| **Pedersen, 2016^64^** | USA | Euthyroid women between 35 to 36 weeks pregnant recruited from a public health obstetrics clinic | 199 | NR |
| **Pinheiro 2013^65^** | Brazil | Women between 32 and 36 weeks pregnant recruited from the antenatal clinics in western Nigeria | 207 | 27 (13) |

**Abbreviations**: NR: Not Reported; UK: United Kingdom; USA: United States of America.

**Supplementary Table 2 References**

1. Aceti F, Aveni F, Baglioni V, Carluccio GM, Colosimo D, Giacchetti N, et al. Perinatal and postpartum depression: from attachment to personality. A pilot study. J Psychopathology. 2012;**18**:328-34.
2. Barnes J, Senior R, MacPherson K. The utility of volunteer home‐visiting support to prevent maternal depression in the first year of life. Child Care Health Dev. 2009**;35**:807-16.
3. Bavle AD, Chandahalli AS, Phatak AS, Rangaiah N, Kuthandahalli SM, Nagendra PN. Antenatal depression in a tertiary care hospital. Indian J Psychol Med. 2016;**38**:31.
4. Beck CT, Gable RK. Comparative analysis of the performance of the Postpartum Depression Screening Scale with two other depression instruments. Nurs Res. 2001;**50**:242-50.
5. Bunevicius A, Kusminskas L, Pop VJ, Pedersen CA, Bunevicius R. Screening for antenatal depression with the Edinburgh Depression Scale. J Psychosom Obstet Gynecol*.* 2009;**30**:238-43.
6. Chaudron LH, Szilagyi PG, Tang W, Anson E, Talbot NL, Wadkins HI, et al. Accuracy of depression screening tools for identifying postpartum depression among urban mothers. Pediatrics. 2010**:125**:e609-17.
7. de Figueiredo FP, Parada AP, Cardoso VC, Batista RF, da Silva AA, Barbieri MA, et al. Postpartum depression screening by telephone: a good alternative for public health and research. Arch Womens Ment Health. 2015;**18**:547-53.
8. Garcia-Esteve L, Ascaso C, Ojuel J, Navarro P. Validation of the Edinburgh postnatal depression scale (EPDS) in Spanish mothers. J Affect Disord. 2003;**75**:71-6.
9. Giardinelli L, Innocenti A, Benni L, Stefanini MC, Lino G, Lunardi C, et al. Depression and anxiety in perinatal period: prevalence and risk factors in an Italian sample. Arch Womens Ment Health. 2012;**15**:21-30.
10. Helle N, Barkmann C, Bartz-Seel J, Diehl T, Ehrhardt S, Hendel A, et al. Very low birth-weight as a risk factor for postpartum depression four to six weeks postbirth in mothers and fathers: Cross-sectional results from a controlled multicentre cohort study. J Affect Disord. 2015;**180:**154-61.
11. Hickey AR, Boyce PM, Ellwood D, Morris-Yates AD. Early discharge and risk for postnatal depression. Med J Aust*.* 1997;**167**:244-7.
12. Howard LM, Ryan EG, Trevillion K, Anderson F, Bick D, Bye A, et al. Accuracy of the Whooley questions and the Edinburgh Postnatal Depression Scale in identifying depression and other mental disorders in early pregnancy. Br J Psychiatry. 2018;**212**:50-6.
13. Leonardou AA, Zervas YM, Papageorgiou CC, Marks MN, Tsartsara EC, Antsaklis A, et al. Validation of the Edinburgh Postnatal Depression Scale and prevalence of postnatal depression at two months postpartum in a sample of Greek mothers. J Reprod Infant Psychol. 2009;**27**:28-39.
14. Navarro P, Ascaso C, Garcia-Esteve L, Aguado J, Torres A, Martín-Santos R. Postnatal psychiatric morbidity: a validation study of the GHQ-12 and the EPDS as screening tools. Gen Hosp Psychiatry. 2007;**29**:1-7.
15. Nakić Radoš, Tadinac M, Herman R. Validation study of the Croatian version of the Edinburgh Postnatal Depression Scale (EPDS). Suvrem Psihol*.* 2013;**16**:203-18.
16. Phillips J, Charles M, Sharpe L, Matthey S. Validation of the subscales of the Edinburgh Postnatal Depression Scale in a sample of women with unsettled infants. J Affect Disord. 2009;**118**:101-12.
17. Prenoveau J, Craske M, Counsell N, West V, Davies B, Cooper P, et al. Postpartum GAD is a risk factor for postpartum MDD: the course and longitudinal relationships of postpartum GAD and MDD. Depress Anxiety. 2013;**30**:506-14.
18. Robertson-Blackmore E, Putnam FW, Rubinow DR, Matthieu M, Hunn JE, Putnam KT, Moynihan JA, O'Connor TG. Antecedent trauma exposure and risk of depression in the perinatal period. J Clin Psychiatry*.* 2013;**74:**e942-8.
19. Rochat TJ, Tomlinson M, Newell ML, Stein A. Detection of antenatal depression in rural HIV-affected populations with short and ultrashort versions of the Edinburgh Postnatal Depression Scale (EPDS). Arch Womens Ment Health. 2013;**16**:401-10.
20. Siu BW, Leung SS, Ip P, Hung SF, O'Hara MW. Antenatal risk factors for postnatal depression: a prospective study of Chinese women at maternal and child health centres. BMC Psychiatry. 2012;**12**:22.
21. Stewart RC, Umar E, Tomenson B, Creed F. Validation of screening tools for antenatal depression in Malawi—A comparison of the Edinburgh Postnatal Depression Scale and Self Reporting Questionnaire. J Affect Disord. 2013;**150**:1041-7.
22. Tandon SD, Cluxton-Keller F, Leis J, Le HN, Perry DF. A comparison of three screening tools to identify perinatal depression among low-income African American women. J Affect Disord. 2012;**136**:155-62.
23. Tendais I, Costa R, Conde A, Figueiredo B. Screening for depression and anxiety disorders from pregnancy to postpartum with the EPDS and STAI. Span J of Psychol. 2014;**17**:E7.
24. Töreki A, Andó B, Keresztúri A, Sikovanyecz J, Dudas RB, Janka Z, et al. The Edinburgh Postnatal Depression Scale: translation and antepartum validation for a Hungarian sample. Midwifery. 2013;**29**:308-15.
25. Töreki A, Andó B, Dudas RB, Dweik D, Janka Z, Kozinszky Z, Keresztúri A. Validation of the Edinburgh Postnatal Depression Scale as a screening tool for postpartum depression in a clinical sample in Hungary. Midwifery. 2014;**30**:911-8.
26. Tran TD, Tran T, La B, Lee D, Rosenthal D, Fisher J. Screening for perinatal common mental disorders in women in the north of Vietnam: a comparison of three psychometric instruments. J Affect Disord. 2011;**133**:281-93.
27. Turner K, Piazzini A, Franza A, Marconi AM, Canger R, Canevini MP. Epilepsy and postpartum depression. Epilepsia*.* 2009;**50**:24-7.
28. Vega-Dienstmaier JM, Mazzotti GS, Campos MS. Validation of a Spanish version of the Edinburgh postnatal depression scale. Actas Esp Psiquiatr. 2002;**30**:106-11.
29. Fisher JR, Wynter KH, Rowe HJ. Innovative psycho-educational program to prevent common postpartum mental disorders in primiparous women: a before and after controlled study*.* BMC Public Health. 2010;**10**:432.
30. Rowe HJ, Fisher JR, Loh WM. The Edinburgh Postnatal Depression Scale detects but does not distinguish anxiety disorders from depression in mothers of infants. Arch Womens Ment Health. 2008;**11**:103-8.
31. Yonkers KA, Smith MV, Forray A, Epperson CN, Costello D, Lin H, Belanger K. Pregnant women with posttraumatic stress disorder and risk of preterm birth. JAMA Psychiatry. 2014;**71**:897-904.
32. Alvarado R, Jadresic E, Guajardo V, Rojas G. First validation of a Spanish-translated version of the Edinburgh postnatal depression scale (EPDS) for use in pregnant women. A Chilean study. Arch Womens Ment Health. 2015;**18**:607-12.
33. Alvarado-Esquivel C, Sifuentes-Alvarez A, Salas-Martinez C, Martínez-García S. Validation of the Edinburgh Postpartum Depression Scale in a population of puerperal women in Mexico. Clin Pract Epidemiol Ment Health. 2006;**2**:33.
34. Alvarado-Esquivel C, Sifuentes-Alvarez A, Salas-Martinez C. Detection of mental disorders other than depression with the Edinburgh Postnatal Depression Scale in a sample of pregnant women in northern Mexico. Mental Illness*.* 2016;**8**:6021.
35. Bakare MO, Okoye JO, Obindo JT. Introducing depression and developmental screenings into the National Programme on Immunization (NPI) in southeast Nigeria: an experimental cross-sectional assessment. Gen Hosp Psychiatry. 2014;**36**:105-12.
36. Couto TC, Brancaglion MY, Cardoso MN, Protzner AB, Garcia FD, Nicolato R, Aguiar RA, Leite HV, Corrêa H. What is the best tool for screening antenatal depression? J Affect Disord. 2015;**178**:12-7.
37. Comasco E, Gulinello M, Hellgren C, Skalkidou A, Sylven S, Sundström-Poromaa I. Sleep duration, depression, and oxytocinergic genotype influence prepulse inhibition of the startle reflex in postpartum women. Eur Neuropsychopharmacol. 2016;**26:**767-76.
38. Eapen V, Johnston D, Apler A, Rees S, Silove DM. Adult separation anxiety during pregnancy and its relationship to depression and anxiety. J Perinat Med. 2013;**41**:159-63.
39. Fernandes MC, Srinivasan K, Stein AL, Menezes G, Sumithra RS, Ramchandani PG. Assessing prenatal depression in the rural developing world: a comparison of two screening measures. Arch Womens Ment Health. 2011;**14**:209-16.
40. Figueira P, Corrêa H, Malloy-Diniz L, Romano-Silva MA. Edinburgh Postnatal Depression Scale for screening in the public health system. Rev Saude Publica. 2009;**43**:79-84.
41. Imbula BE, Okitundu EL, Mampunza SM. Postpartum depression in Kinshasa (DR Congo): prevalence and risk factors. Med Sante Trop. 2012;**22**:379-84.
42. Khalifa DS, Glavin K, Bjertness E, Lien L. Postnatal depression among Sudanese women: prevalence and validation of the Edinburgh Postnatal Depression Scale at 3 months postpartum. Health Care Women Int. 2015;**7**:677-84.
43. Roomruangwong C, Kanchanatawan B, Sirivichayakul S, Maes M. Antenatal depression and hematocrit levels as predictors of postpartum depression and anxiety symptoms. Psychiatry Res. 2016;**238:**211-7.
44. Su KP, Chiu TH, Huang CL, Ho M, Lee CC, Wu PL, et al. Different cutoff points for different trimesters? The use of Edinburgh Postnatal Depression Scale and Beck Depression Inventory to screen for depression in pregnant Taiwanese women. Gen Hosp Psychiatry. 2007;**29**:436-41.
45. Thiagayson P, Krishnaswamy G, Lim ML, Sung SC, Haley CL, Fung DS, et al. Depression and anxiety in Singaporean high-risk pregnancies—prevalence and screening. Gen Hosp Psychiatry. 2013;**35**:112-6.
46. Usuda K, Nishi D, Makino M, Tachimori H, Matsuoka Y, Sano Y, et al. Prevalence and related factors of common mental disorders during pregnancy in Japan: a cross-sectional study. Biopsychosoc Med. 2016;**10**:17.
47. Aydin N, Inandi T, Yigit A, Hodoglugil NN. Validation of the Turkish version of the Edinburgh Postnatal Depression Scale among women within their first postpartum year. Soc Psychiatry Psychiatr Epidemiol. 2004;**39**:483-6.
48. Banti S, Mauri M, Oppo A, Borri C, Rambelli C, Ramacciotti D, et al. From the third month of pregnancy to 1 year postpartum. Prevalence, incidence, recurrence, and new onset of depression. Results from the perinatal depression-research & screening unit study. Compr Psychiatry. 2011;**52**:343-51.
49. Brodey BB, Goodman SH, Baldasaro RE, Brooks-DeWeese A, Wilson ME, Brodey ISB, Doyle NM. Development of the Perinatal Depression Inventory (PDI)-14 using item response theory: a comparison of the BDI-II, EPDS, PDI, and PHQ-9. Arch Womens Ment Health. 2016;**19**:307-16.
50. Chibanda D, Mangezi W, Tshimanga M, Woelk G, Rusakaniko P, Stranix-Chibanda L, et al. Validation of the Edinburgh Postnatal Depression Scale among women in a high HIV prevalence area in urban Zimbabwe. Arch Womens Ment Health. 2010;**13**:201-6.
51. Gausia K, Fisher C, Algin S, Oosthuizen J. Validation of the Bangla version of the Edinburgh Postnatal Depression Scale for a Bangladeshi sample. J Reprod Infant Psychol. 2007;**25**:308-15.
52. Gorman LL, O'Hara MW, Figueiredo B, Hayes S, Jacquemain F, Kammerer MH, et al. Adaptation of the structured clinical interview for DSM-IV disorders for assessing depression in women during pregnancy and post-partum across countries and cultures. Br J Psychiatry Suppl. 2004;**46**:s17.
53. Li L, Liu F, Zhang H, Wang L, Chen X. Chinese version of the Postpartum Depression Screening Scale: translation and validation. Nurs Res. 2011;**60**:231-9.
54. Moses-Kolko EL, Price JC, Wisner KL, Hanusa BH, Meltzer CC, Berga SL, et al. Postpartum and depression status are associated with lower [^11^C]raclopride BP_ND_ in Reproductive-Age Women. Neuropsychopharmacol. 2012;**37**:1422-32.
55. Stuebe AM, Grewen K, MeltzerBrody S. Association between maternal mood and oxytocin response to breastfeeding. J Womens Health. 2013;**22**:352.
56. Bergink V, Kooistra L, Lambregtse-van den Berg MP, Wijnen H, Bunevicius R, van Baar A, Pop V. Validation of the Edinburgh Depression Scale during pregnancy. J Psychosom Res. 2011;**70**:385-9.
57. Mahmud WM, Awang A, Mohamed MN. Revalidation of the Malay Version of the Edinburgh Postnatal Depression Scale (EPDS) Among Malay Postpartum Women Attending the Bakar Bata Health Center in Alor Setar, Kedah, North West Of Peninsular Malaysia. Malays J Med Sci. 2003;**10**:71-5.
58. Adewuya AO, Ola BA, Dada AO, Fasoto OO. Validation of the Edinburgh Postnatal Depression Scale as a screening tool for depression in late pregnancy among Nigerian women. J Psychosom Obstet Gynecol. 2006;**27**:267-72.
59. Adouard F,​ Glangeaud-Freudenthal NM, ​Golse B. Validation of the Edinburgh Postnatal Depression Scale (EPDS) in a sample of women with high-risk pregnancies in France. Arch Womens Ment Health. 2005;**8**:89-95.
60. Agoub M, Moussaoui D, Battas O. Prevalence of postpartum depression in a Moroccan sample. Arch Womens Ment Health. 2005;**8**:37-43.
61. Benvenuti P, Ferrara M, Niccolai C, Valoriani V, Cox JL. The Edinburgh Postnatal Depression Scale: validation for an Italian sample. J Affective Disord. 1999;**53**:137-41.
62. Berle JØ, Aarre TF, Mykletun A, Dahl AA, Holsten F. Screening for postnatal depression. Validation of the Norwegian version of the Edinburgh Postnatal Depression Scale, and assessment of risk factors for postnatal depression. J Affective Disord. 2003;**76**:151-6.
63. Christl B, Reilly N, Smith M, Sims D, Chavasse F, Austin MP. The mental health of mothers of unsettled infants: is there value in routine psychosocial assessment in this context? Arch Womens Ment Health. 2013;**16**:391-9.
64. Pedersen C, Leserman J, Garcia N, Stansbury M, Meltzer-Brody S, Johnson J. Late pregnancy thyroid-binding globulin predicts perinatal depression. Psychoneuroendocrinology. 2016;**65**:84-93.
65. Pinheiro RT, Coelho FM, Silva RA, Pinheiro KA, Oses JP, Quevedo Lde A, et al. Association of a serotonin transporter gene polymorphism (5-HTTLPR) and stressful life events with postpartum depressive symptoms: a population-based study. J Psychosom Obstet Gynecol. 2013;**34**:29-33.

**Supplementary Table 3. Number and Proportion of Participants with Major Depression at each EPDS Score for the Structured Clinical Interview for DSM Disorders, Composite International Diagnostic Interview, and Mini Neurospsychiatric Diagnostic Interview**

|  | **SCID** | | **CIDI** | | **MINI** | |
| --- | --- | --- | --- | --- | --- | --- |
| **EPDS Score** | **N**  **Total** | **N (%)**  **Major Depression** | **N**  **Total** | **N (%)**  **Major Depression** | **N**  **Total** | **N (%)**  **Major Depression** |
| **0** | 637 | 7 (1) | 385 | 1 (0) | 198 | 1 (1) |
| **1** | 483 | 6 (1) | 299 | 5 (2) | 157 | 1 (1) |
| **2** | 607 | 5 (1) | 245 | 5 (2) | 204 | 4 (2) |
| **3** | 559 | 4 (1) | 243 | 4 (2) | 167 | 3 (2) |
| **4** | 522 | 12 (2) | 231 | 5 (2) | 223 | 3 (1) |
| **5** | 491 | 14 (3) | 197 | 5 (3) | 202 | 5 (2) |
| **6** | 481 | 22 (5) | 194 | 4 (2) | 187 | 14 (7) |
| **7** | 471 | 31 (7) | 138 | 4 (3) | 148 | 7 (5) |
| **8** | 437 | 31 (7) | 144 | 8 (6) | 148 | 14 (9) |
| **9** | 436 | 33 (8) | 130 | 3 (2) | 135 | 15 (11) |
| **10** | 333 | 36 (11) | 119 | 6 (5) | 118 | 8 (7) |
| **11** | 294 | 60 (20) | 107 | 17 (16) | 103 | 30 (29) |
| **12** | 271 | 74 (27) | 84 | 10 (12) | 93 | 16 (17) |
| **13** | 234 | 75 (32) | 76 | 10 (13) | 79 | 25 (32) |
| **14** | 189 | 74 (39) | 77 | 17 (22) | 52 | 24 (46) |
| **15** | 175 | 84 (48) | 63 | 11 (17) | 68 | 31 (46) |
| **16** | 127 | 71 (56) | 57 | 15 (26) | 65 | 30 (46) |
| **17** | 118 | 69 (58) | 39 | 13 (33) | 32 | 14 (44) |
| **18** | 99 | 62 (63) | 42 | 13 (31) | 34 | 17 (50) |
| **19** | 79 | 54 (68) | 24 | 13 (54) | 29 | 15 (52) |
| **20** | 45 | 33 (73) | 17 | 4 (24) | 23 | 19 (83) |
| **21** | 53 | 40 (75) | 15 | 6 (40) | 24 | 16 (67) |
| **22** | 43 | 39 (91) | 6 | 4 (67) | 10 | 6 (60) |
| **23** | 27 | 22 (81) | 7 | 6 (86) | 7 | 5 (71) |
| **24** | 26 | 23 (88) | 1 | 1 (100) | 10 | 7 (70) |
| **25** | 13 | 9 (69) | 2 | 0 (0) | 8 | 6 (75) |
| **26** | 10 | 10 (100) | 2 | 1 (50) | 2 | 1 (50) |
| **27** | 6 | 4 (67) | 2 | 1 (50) | 4 | 4 (100) |
| **28** | 7 | 7 (100) | 1 | 1 (100) | 1 | 0 (0) |
| **29** | 5 | 5 (100) | 1 | 1 (100) | 1 | 1 (100) |
| **30** | 1 | 1 (100) | 0 | 0 (0) | 0 | 0 (0) |
| **Total** | 7279 | 1017 (14) | 2948 | 194 (7) | 2532 | 342 (14) |

**Abbreviations**: CIDI: Composite International Diagnostic Interview; EPDS: Edinburgh Postnatal Depression Scale; MINI: Mini Neurospsychiatric Diagnostic Interview; SCID: Structured Clinical Interview for DSM Disorders

**Supplementary Table 4a. Estimates of Fixed Effects from Model Comparing the Mini Neurospsychiatric Diagnostic Interview to the Composite International Diagnostic Interview**

| **Fixed effect** | **Estimate** | **SD** | **P-value** |
| --- | --- | --- | --- |
| Intercept | -5.810 | 0.607 | <0.001 |
| EPDS | 0.262 | 0.011 | <0.001 |
| Age (years) | 0.004 | 0.010 | 0.689 |
| Country Human Development Index (reference = very high) |  |  |  |
| High | -1.188 | 0.477 | 0.013 |
| Low to medium | 0.287 | 0.561 | 0.609 |
| Pregnancy status (reference = pregnant) |  |  |  |
| Postpartum | 0.349 | 0.441 | 0.429 |
| Diagnostic interview (reference = CIDI) |  |  |  |
| MINI | 1.314 | 0.573 | 0.022 |

**Abbreviations**: CIDI: Composite International Diagnostic Interview; EPDS: Edinburgh Postnatal Depression Scale; MINI: Mini Neurospsychiatric Diagnostic Interview; SD: standard deviation

**Supplementary Table 4b. Estimates of Fixed Effects from Model Comparing the Composite International Diagnostic Interview to the Structured Clinical Interview for DSM Disorders**

| **Fixed effect** | **Estimate** | **SD** | **P-value** |
| --- | --- | --- | --- |
| Intercept | -5.210 | 0.382 | <0.001 |
| EPDS | 0.312 | 0.009 | <0.001 |
| Age (years) | -0.001 | 0.007 | 0.868 |
| Country Human Development Index (reference = very high) |  |  |  |
| High | 0.201 | 0.668 | 0.764 |
| Low to medium | 0.792 | 0.612 | 0.195 |
| Pregnancy status (reference = pregnant) |  |  |  |
| Postpartum | 0.108 | 0.257 | 0.675 |
| Diagnostic interview (reference = SCID) |  |  |  |
| CIDI | -1.076 | 0.699 | 0.124 |

**Abbreviations**: CIDI: Composite International Diagnostic Interview; EPDS: Edinburgh Postnatal Depression Scale; SCID: Structured Clinical Interview for DSM Disorders; SD: standard deviation

**Supplementary Table 4c. Estimates of Fixed Effects from Model Comparing the Mini Neurospsychiatric Diagnostic Interview to the Structured Clinical Interview for DSM Disorders**

| **Fixed effect** | **Estimate** | **SD** | **P-value** |
| --- | --- | --- | --- |
| Intercept | -5.409 | 0.362 | <0.001 |
| EPDS | 0.332 | 0.009 | <0.001 |
| Age (years) | -0.001 | 0.007 | 0.872 |
| Country Human Development Index (reference = very high) |  |  |  |
| High | -0.599 | 0.475 | 0.208 |
| Low to medium | 0.749 | 0.457 | 0.101 |
| Pregnancy status (reference = pregnant) |  |  |  |
| Postpartum | 0.210 | 0.237 | 0.377 |
| Diagnostic interview (reference = SCID) |  |  |  |
| MINI | -0.095 | 0.387 | 0.806 |

**Abbreviations**: EPDS: Edinburgh Postnatal Depression Scale; MINI: Mini Neurospsychiatric Diagnostic Interview; SCID: Structured Clinical Interview for DSM Disorders; SD: standard deviation

**Supplementary Table 4d. Estimates of Fixed Effects from Model Comparing the Mini Neurospsychiatric Diagnostic Interview (MINI) to the Composite International Diagnostic Interview, Including an Interaction between MINI and Depressive Symptom Severity based on the Edinburgh Postnatal Depression Scale**

| **Fixed effect** | **Estimate** | **SD** | **P-value** |
| --- | --- | --- | --- |
| Intercept | -5.339 | 0.642 | <0.001 |
| EPDS | 0.226 | 0.015 | <0.001 |
| Age (years) | 0.002 | 0.010 | 0.874 |
| Country Human Development Index (reference = very high) |  |  |  |
| High | -1.340 | 0.506 | 0.008 |
| Low to medium | 0.192 | 0.594 | 0.747 |
| Pregnancy status (reference = pregnant) |  |  |  |
| Postpartum | 0.423 | 0.466 | 0.363 |
| Diagnostic interview (reference = CIDI) |  |  |  |
| MINI | 0.543 | 0.644 | 0.399 |
| MINI*EPDS | 0.071 | 0.021 | <0.001 |

**Abbreviations**: CIDI: Composite International Diagnostic Interview; EPDS: Edinburgh Postnatal Depression Scale; MINI: Mini Neurospsychiatric Diagnostic Interview; SD: standard deviation

**Supplementary Table 4e. Estimates of Fixed Effects from Model Comparing the Composite International Diagnostic Interview (CIDI) to the Structured Clinical Interview for DSM Disorders, Including an Interaction between CIDI and Depressive Symptom Severity based on the Edinburgh Postnatal Depression Scale**

| **Fixed effect** | **Estimate** | **SD** | **P-value** |
| --- | --- | --- | --- |
| Intercept | -5.521 | 0.400 | <0.001 |
| EPDS | 0.347 | 0.011 | <0.001 |
| Age (years) | -0.005 | 0.008 | 0.526 |
| Country Human Development Index (reference = very high) |  |  |  |
| High | 0.186 | 0.712 | 0.794 |
| Low to medium | 0.831 | 0.651 | 0.202 |
| Pregnancy status (reference = pregnant) |  |  |  |
| Postpartum | 0.117 | 0.267 | 0.662 |
| Diagnostic interview (reference = SCID) |  |  |  |
| CIDI | 0.443 | 0.767 | 0.563 |
| CIDI*EPDS | -0.123 | 0.018 | <0.001 |

**Abbreviations**: CIDI: Composite International Diagnostic Interview; EPDS: Edinburgh Postnatal Depression Scale; SCID: Structured Clinical Interview for DSM Disorders; SD: standard deviation

**Supplementary Table 4f. Estimates of Fixed Effects from Model Comparing the the Mini Neurospsychiatric Diagnostic Interview (MINI) to the Structured Clinical Interview for DSM Disorders, Including an Interaction between MINI and Depressive Symptom Severity based on the Edinburgh Postnatal Depression Scale**

| **Fixed effect** | **Estimate** | **SD** | **P-value** |
| --- | --- | --- | --- |
| Intercept | -5.575 | 0.370 | <0.001 |
| EPDS | 0.346 | 0.010 | <0.001 |
| Age (years) | -0.001 | 0.007 | 0.860 |
| Country Human Development Index (reference = very high) |  |  |  |
| High | -0.539 | 0.477 | 0.258 |
| Low to medium | 0.771 | 0.459 | 0.093 |
| Pregnancy status (reference = pregnant) |  |  |  |
| Postpartum | 0.203 | 0.239 | 0.396 |
| Diagnostic interview (reference = SCID) |  |  |  |
| MINI | 0.478 | 0.444 | 0.282 |
| MINI*EPDS | -0.049 | 0.019 | 0.009 |

**Abbreviations**: EPDS: Edinburgh Postnatal Depression Scale; MINI: Mini Neurospsychiatric Diagnostic Interview; SCID: Structured Clinical Interview for DSM Disorders; SD: standard deviation
